# Matrigel patterning reflects multicellular contractility

**DOI:** 10.1371/journal.pcbi.1007431

**Published:** 2019-10-25

**Authors:** Előd Méhes, Beáta Biri-Kovács, Dona G. Isai, Márton Gulyás, László Nyitray, András Czirók

**Affiliations:** 1 Department of Biological Physics, Eotvos Lorand University, Budapest, Hungary; 2 Department of Biochemistry, Eotvos Lorand University, Budapest, Hungary; 3 Department of Anatomy & Cell Biology, University of Kansas Medical Center, Kansas City, Kansas, United States of America; Oxford, UNITED KINGDOM

## Abstract

Non-muscle myosin II (NMII)-induced multicellular contractility is essential for development, maintenance and remodeling of tissue morphologies. Dysregulation of the cytoskeleton can lead to birth defects or enable cancer progression. We demonstrate that the Matrigel patterning assay, widely used to characterize endothelial cells, is a highly sensitive tool to evaluate cell contractility within a soft extracellular matrix (ECM) environment. We propose a computational model to explore how cell-exerted contractile forces can tear up the cell-Matrigel composite material and gradually remodel it into a network structure. We identify measures that are characteristic for cellular contractility and can be obtained from image analysis of the recorded patterning process. The assay was calibrated by inhibition of NMII activity in A431 epithelial carcinoma cells either directly with blebbistatin or indirectly with Y27632 Rho kinase inhibitor. Using Matrigel patterning as a bioassay, we provide the first functional demonstration that overexpression of S100A4, a calcium-binding protein that is frequently overexpressed in metastatic tumors and inhibits NMIIA activity by inducing filament disassembly, effectively reduces cell contractility.

## Author summary

Sensing and exerting forces is a fundamental aspect of tissue organization. We demonstrate that contractile cells form an intricate network structure when placed in a pliable culture environment, a phenomenon often associated with vascular networks and is being actively used to characterize endothelial cells in culture. We propose a computational model that operates with mechanical stresses, plastic deformation and material failure within the cell-extracellular matrix composite to explain the patterning process. In addition to re-interpret a decades-old tool of experimental cell biology, our work suggests a potentially high throughput computational assay to characterize cellular contractility within a soft ECM environment.

## Introduction

Cells use contractile forces, generated and regulated by cytoskeletal proteins, to maintain a structured multicellular tissue [1]. Cell-cell adhesion and tissue surface tension are contractility-related phenomena that have key roles in providing tissue integrity and driving morphogenesis [2–4]. Multicellular contractility plays a prominent role in various physiological and developmental processes [5]. Some of the best characterized examples come from embryonic morphogenesis events where coordinated multicellular contractility is the major driver of cell movements and subsequent cell differentiation. During the formation of the neural tube in vertebrates, the bending of the neural plate epithelium is driven by coordinated apical constriction of cells, which is regulated by Rho kinase recruitment and local activation of non-muscle myosin II (NMII) at cell apexes [6–9]. Another extensively studied multicellular contractility-driven process is the early embryonic development of insects. In Drosophila the germ band develops into the segmented trunk of the embryo. This convergent extension process involves cell intercalation, mediated by NMII-dependent cortical tension of germ band cells [10–12]. In cell culture wound healing experiments cell contractility exhibits a non-uniform spatial distribution, suggested to coordinate two-dimensional collective cell migration [13]. Contractility also has substantial roles in cancer progression and metastasis [14, 15].

At the molecular level cell contractility is driven by the activity of non-muscle myosin II (NMII), organized into bipolar minifilaments sliding along actin filaments of the cytoskeleton [16]. Myosins are actin-binding motor proteins that use the energy of ATP to produce forces required not only in muscle contraction, but also in processes like cytokinesis, cell adhesion and migration. Non-muscle myosin II, in particular, is a molecular motor abundant in practically all animal cell types [17]. It is composed of two heavy chains, two essential and two regulatory light chains. The heavy chains fold into two globular head domains containing binding sites for ATP and actin, followed by two neck regions that function as lever arms during force generation, and finally into a long dimeric coiled-coil tail and a nonhelical tailpiece. The neck region level arms are stabilized through forming a complex with the NMII light chains. Usually, 28 NMII molecules assemble into bipolar “minifilaments” through electrostatic interactions of the coiled-coil tails. Single NMII molecules and even dimers cannot move actin filaments, the onset of this activity requires at least a tetrameric state. NMII can exert intercellular forces at cell junctions as well as traction forces transmitted to the extracellular matrix substrate.

NMII activity is regulated by various means, including the binding of protein interaction partners. One of the binding partners of NMII is the metastasis-associated protein S100A4 (also called metastasin). Members of the S100 protein family are small, calcium-binding proteins that exert their function mainly by forming complexes with their regulatory targets. S100A4 is one of the most studied members of the family as it is frequently overexpressed in metastatic tumors [18]. The molecular interaction between S100A4 and a particular NMII isoform, NMIIA, is well studied [19]. S100A4 binds to a region that overlaps the coiled-coil assembly competence domain (responsible for filament formation) and the C-terminal non-helical tailpiece [20–22]. Thus, the presence of S100A4 disrupts existing myosin filaments and also prevents their assembly [23]. Hence, S100A4 can control cell contractility by regulating the dynamic assembly of NMIIA.

The current tool to assay cell contractility is traction force microscopy, which characterizes cell-exerted forces by measuring deformations of an elastic substrate [24–26]. Initially applied for single cells, this method has been extended to monolayer cultures yielding a spatio-temporal map of traction stresses across a multicellular domain extending several millimeters [27]. Traction force microscopy, however, is a computationally intensive and complex measurement, which requires the preparation of a suitably homogeneous and well-defined elastic substrate with known constitutive equation and material parameters [28]. Preparation of such uniform, well defined elastic substrate is also challenging for soft substrates that tend to polymerize in a spatially inhomogeneous structure [29]. Indicative of the inherently low throughput nature of traction force microscopy, authors of this paper were unable to find a single study reporting dose-dependent traction force microscopy data—measurements repeated at multiple concentrations of the tested compound.

Here we demonstrate that a suitable quantification of the well-known Matrigel tube formation assay is highly informative about cell-exerted contractile forces, and can be utilized as a much simpler bioassay than traction force microscopy. We propose a computational model of the corresponding multicellular patterning process and identify measures that are characteristic of cellular contractility and can be obtained by time-lapse image analysis. We validated our model and calibrated the bioassay by direct and indirect small molecule inhibitors of NMII. While this method does not provide spatially resolved contractility maps, it is much easier to perform than traditional traction force microscopy. To demonstrate its applicability we characterize how overexpression of S100A4, an intrinsic regulator of NMIIA filament assembly, modulates the contractility of epithelial carcinoma cells.

## Results

### Matrigel patterning assay

**Fig 1:**
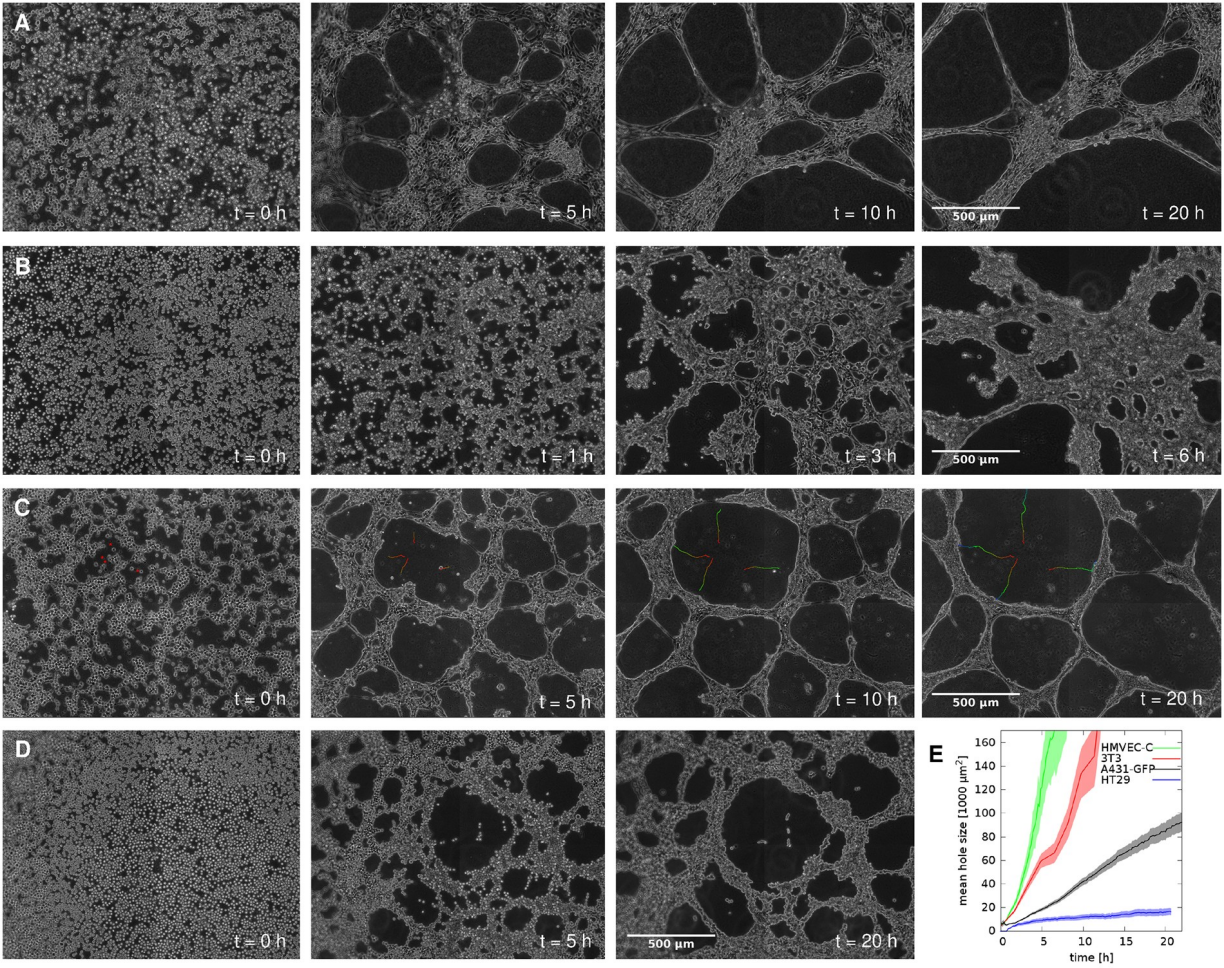
Pattern formation on Matrigel substrate. Several cell types condense into progressively denser clusters, creating thereby a network which delineates cell-free areas (“holes”). Phase-contrast micrographs, recorded at the indicated times after seeding, show HMVEC-C human cardiac microvascular endothelial cells (A), 3T3 fibroblasts (B), A431 human epithelial carcinoma cells (C) and HT29 human colon adenocarcinoma cells (D). The patterning processes are documented in S1–S4 Movies. Trajectories in panel (C) depict the movement of 4 representative features within the Matrigel ECM, marked with red asterisks (*) in the first panel of the time series. Trajectories are color-coded, warmer colors indicate earlier segments. Cells were seeded at ≈ 66% confluence (1600 cells/mm^2^). Scale bar: 500 μm. E: Quantitative evaluation of the Matrigel patterning assay with various cell types. Time dependence of the mean hole size (cell-free area) of the four cell types: HMVEC-C (green), 3T3 (red), A431 (black) and HT29 cells (blue). Error stripes represent SEM, calculated from at least *n* = 3 independent sets of experiments.

When contractile cells are seeded on the surface of a suitably thick Matrigel extracellular matrix (ECM) layer at a subconfluent density, an interconnected network pattern develops within a few hours (Fig 1 and S1–S5 Movies). The pattern is similar irrespective whether the cells are endothelial (Fig 1A), mesenchymal (Fig 1B), or epithelial (Fig 1C and 1D). The network structure contains both cells and ECM, and delimits “holes”, which are polygonal patches devoid of cells. The seeding cell density is an important experimental parameter: isolated cell clusters form if the seeding density is below the percolation threshold, while cultures maintain a confluent monolayer when seeded at identical conditions but at higher cell density (S6 Movie).

As the pattern forms in a few hours, cell proliferation has only a limited role in the process. Similarly, cell death is hardly detectable in our recordings. Thus, we mainly witness the rearrangement of the seeded cells and their Matrigel ECM environment. More detailed analyses of videomicroscopic observations indicate a complete lack of expansion in any part of the structure—hence under these experimental conditions cells do not engage in multicellular sprout formation. Instead, long and narrow clusters eventually break and retract, letting the adjacent holes merge. These rearrangements, as well as the enlargement of holes involve the co-movement of cells and the local ECM as evidenced by tracking recognizable features within the ECM throughout the image sequence (Fig 1C).

Cell-exerted intercellular forces and cell-substrate traction forces are well known to contract the ECM environment [30, 31]. Therefore, we hypothesize that in our experiments holes represent areas where the mechanical integrity of the cell-Matrigel composite material is compromised, and the observed movement around the several “holes” or wound sites is best described as an elastoplastic creep driven by cellular contractile forces. Specifically, for short time scales (minutes) the cell-Matrigel assembly behaves as an elastic (or viscoelastic) solid, but sufficiently large mechanical stress can induce irreversible plastic deformations and breakage over longer time scales (hours).

### Computational model

**Fig 2:**
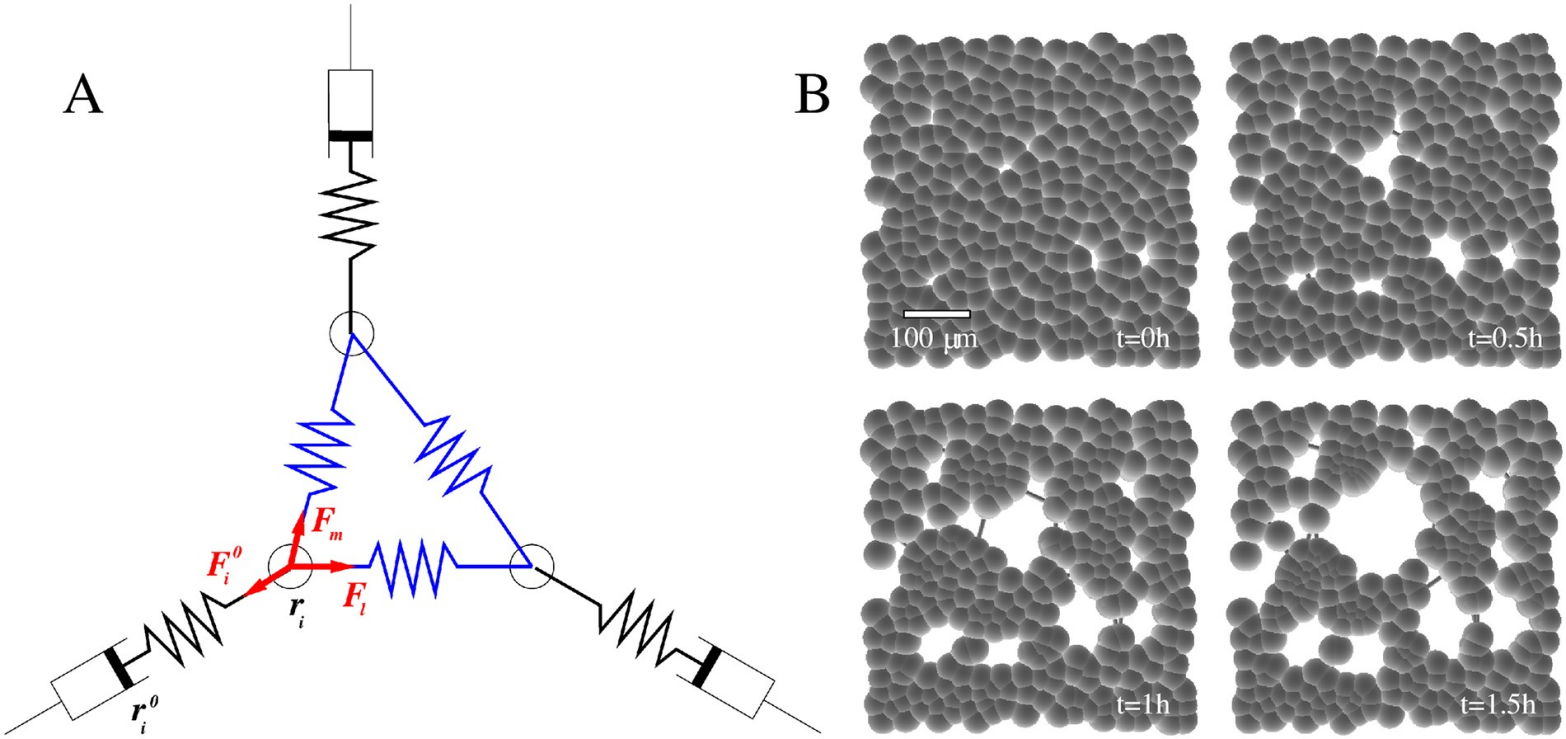
Computational model of contractility-driven plastic patterning. A: Schematic representation of the model. The contractile links (blue) between adjacent particles exert elastic forces **F**_*l*_ and **F**_*m*_ on particle *i*. Adhesion to the substrate is represented by a Mawell element: a dashpot and spring in series (black). The element generates an elastic force $F_{i}^{0}$, which is determined by the relative positions of the particle, **r**_*i*_, and of the dashpot, $r_{i}^{0}$. This latter variable characterizes the ECM microenvironment and can move arbitrary large distances relative to the underlying rigid substrate—our system of reference. B: Time development of a simulated system. Particles are visualized as spheres with a radius of *d*_0_, hence particles touching can establish a mechanical connection according to Eq (11). Mechanical interaction between adjacent particles is transmitted through elastic beams, visible only when the distance between the particles is sufficiently large. *N* = 300 particles were placed within an area of 20*d*_0_ × 20*d*_0_ (500 μm × 500 μm), corresponding to a coverage at 75% confluency. The configuration of particles is shown at the onset of simulation, at 0.5 h, 1 h and 1.5 h as indicated by the labels. The scale bar indicates 100 μm. See also S7 Movie.

While several theories have been proposed to describe the patterning process in terms of cellular contractility and mechanical deformation of the substrate [32–35], the role of mechanical failure and the development of discontinuities have not been addressed. Thus, to understand the particular patterning process in the Matrigel assay, we represented our cell contractility-driven plastic flow hypothesis in a computational model. A previously calibrated particle-and-beam model [36] that explicitly represents intercellular connections and their mechanical load-mediated failure was especially suitable to adapt. Thus, as we describe in detail in the Methods and Models section, we considered cells that are adherent both to the substrate and to each other, and load their adhesion sites with a steady contractile force. Specifically, particles in the model represent cells with their ECM microenvironment, and contractility was modeled by gradually reducing the tension-free length of the beams connecting particles in such a way that particles maintained a pre-determined tension in each link. This particular contractile behavior is selected based on its simplicity, further regulatory mechanisms of cellular contractility can be introduced in future studies. Finally, as a soft Matrigel layer mediates adhesion between the cells and an underlying rigid substrate, we implemented visco-elastic Maxwell-elements to resist movement driven by intercelluluar mechanical forces (Fig 2A).

Model simulations provide a sequence of stochastic alterations in cell-cell connectivity and the corresponding movements by which the configuration restores mechanical equilibrium. Simulations were started from a subconfluent or confluent monolayer state, and for a broad range of parameters the computational rules readily reproduced the multicellular patterning process seen in vitro (Fig 2B). Specifically, fluctuations in particle density were amplified and led to the formation of mechanical discontinuities which grow and coalesce into increasingly larger particle free areas (“holes”). The constant contractility rule does not lead to a steady state and thus the simulation looses its relevance when hole sizes become comparable to the size of the simulated system.

**Fig 3:**
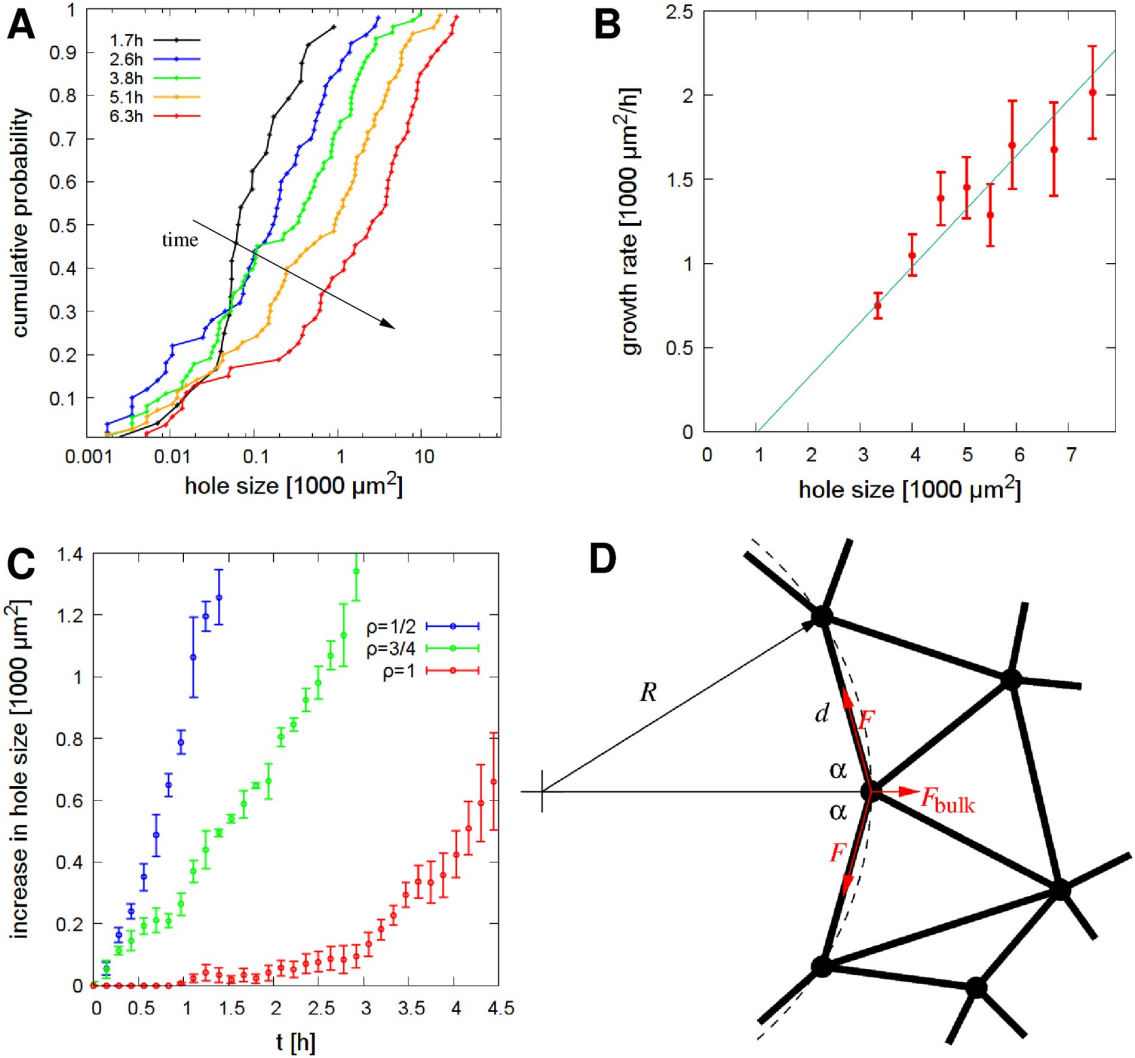
The size of particle free areas (holes) characterize the simulated patterning process. A: Time-dependent cumulative distribution of hole sizes: the ordinate indicates the fraction of holes that are smaller than the value at the abscissa. Initial cell coverage was *ρ* = 75%, distribution functions were compiled from *n* = 4 independent simulation runs. B: Expansion rate of individual holes, as a function of their size. We identified areas that did not merge with adjacent holes during a 30 minute time interval, and determined the change in their size. Error bars represent SEM, binned data is pooled from four independent simulations. The line indicates a linear fit, with a correlation coefficient 0.94. C: Time-dependent increase in the average hole size $\bar{A}\left(t\right)$, compared to the initial condition $\bar{A}\left(t=0\right)$. Blue, green and red lines correspond to simulations started with initial coverages at 50%, 75% and 100% confluency, respectively. The growth rate of holes decreases with increasing initial confluency. Error bars represent SEM, calculated from four independent simulations. D: Schematic representation of particles at a boundary of a circular hole of radius *R*, and the contractile forces acting on a particle in mechanical equilibrium. The contractile force between adjacent particles at the boundary is *F*, while the net contractile force pulling from the bulk is *F*_b*ulk*_.

We characterized the patterning process by calculating the time-dependent distribution of hole sizes (Fig 3A). The distribution shifts to larger values in time, but a population of small holes remains reflecting the ongoing nucleation process. The majority (80%) of holes maintain an approximately lognormal distribution as larger holes grow faster than small ones (Fig 3B). Accordingly, pre-existing larger holes grow more than the smaller ones arising spontaneously within confluent monolayers—and thus patterning becomes faster in cultures with smaller cell density (Fig 3C). In particular, a 50% confluent initial condition yields an expansion twice as fast as the expansion in a simulation with 75% initial confluency—indicated by the time-dependence of the average hole sizes, $\bar{A}\left(t\right)$.

The positive correlation between hole size and expansion rate can be understood by a simplified analysis of model assumptions. Within the model, the force driving hole expansion is determined by the specific balance of tensile forces acting at the boundary (Fig 3D). As there are no forces pulling from an empty area, the contractility of the bulk is balanced by an increased elastic tension *F* along the boundary. If the angle between two links defining the boundary is 2*α*, then the normal component of the forces exerted by boundary links—balancing the net pulling force *F*_b*ulk*_ from the bulk—is 2*F* cos *α*. From geometric considerations the angle *α*, the radius *R* of the hole and the typical distance between particles, *d* are related as 2*R* cos *α* = *d*. Hence, the condition for mechanical equilibrium is

$$F=F_{\text{b}ulk}R/d.$$ (1)

As a crude approximation, we can consider *F*_b*ulk*_ as a constant value set by the contractility homeostasis rule. The model exhibits plastic behavior like creep flow and necking under mechanical load above the yield stress [36], hence large enough tensile forces *F* will gradually increase the length of the boundary by recruiting particles from the bulk. This mechanism also limits the variability of the interparticle distance *d*. As in the case of a bubble its radius grows with its surface tension for a given internal pressure, steady *d* and *F*_b*ulk*_ values in Eq (1) indicate a proportionality between the elastic tensile forces at the boundary, *F*, and the hole size *R*. Furthermore, for the rate of perimeter expansion we expect a linear creep response as

$$dR/dt∼\{\begin{array}{cc} F-F_{\text{m}in}∼R-R_{\text{m}in} & \text{for}\;F>F_{\text{m}in}\\ 0 & \text{otherwise,} \end{array}$$ (2)

where *F*_m*in*_ is the yield stress—the minimal tensile force transmitted by the links that can still induce plastic rearrangement of the particles. The forces *F* and *F*_m*in*_ can be translated to radii *R* and *R*_m*in*_ using relation (1). Similarly, for the area of the hole, *A*, in the *R* ≳ *R*_m*in*_ regime we obtain *dA*/*dt* ∼ *RdR*/*dt* ∼ *R*^2^ − *RR*_m*in*_ ∼ *A* − *A*_m*in*_, qualitatively matching the approximate exponential growth seen in Fig 3B.

**Fig 4:**
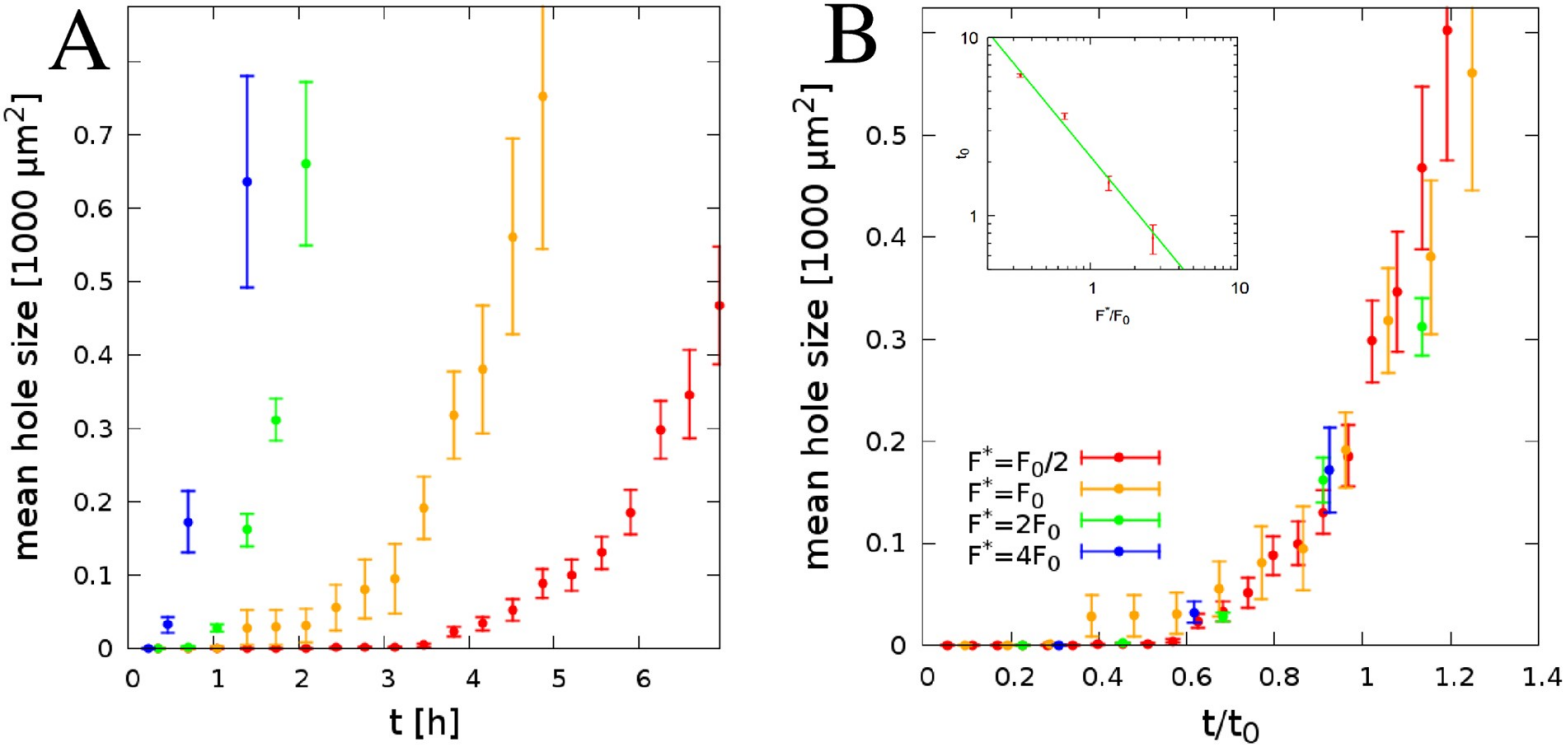
The effect of target contractility F* on simulated pattern formation. A: Time dependence of the average cell-free area size $\bar{A}\left(t\right)$. Blue, green orange and red lines correspond to simulations performed with F*/*F*_0_ values of 4, 2, 1 and 1/2, respectively. Simulations were started from a confluent state (the red curve in Fig 3 matches the orange curve in this figure), thus *ρ* = 1 and $\bar{A}\left(t=0\right)=0$. Error bars represent SEM calculated from *n* = 4 independent simulations. B: The data in panel A collapse to a single curve after scaling the time by an appropriate factor *t*_0_ as $\bar{A}\left(t/t_{0}\right)$. The characteristic time *t*_0_ decreases with F* as *t*_0_ ∼ 1/F* (inset).

Simulations also allow to explore how the targeted magnitude of cell-generated tension, F*, affects the patterning dynamics (Fig 4). As intercellular tension increases the frequency of link removal events, it speeds up the patterning process. Conversely, according to the model, the rate of hole size expansion is indicative of intercellular contractility. As Fig 4B indicates, the $\bar{A}\left(t\right)$ curves, each characteristic for a distinct value of parameter F*, collapse after scaling the time by an appropriate factor *t*_0_ as $\bar{A}\left(t/t_{0}\right)$. Thus, we identify *t*_0_ as the characteristic timescale of the pattern formation and find

$$t_{0}∼1/F^{*},$$ (3)

as shown in the inset of Fig 4B.

The time course of patterning, as characterized by $\bar{A}\left(t\right)$ in Figs 3 and 4, is approximately an exponential and thus exhibits a lag time when no macroscopic holes are present. The emergence of a lag time or a stable confluent monolayer (S6 Movie) is consistent with the presence of a threshold (yield) stress (2) in the sense that the relation (2) predicts no expansion for holes smaller than a critical size. In confluent monolayers discontinuities arise by stochastic events, not described by the plastic creep response (2).

### Validating model predictions by quantitative analysis of experiments

To better characterize the Matrigel patterning bioassay and to validate the computational model, we analyzed time-lapse microscopic images of the patterning process for a variety of cell types including HMVEC-C endothelial cells, A431 epithelial carcinoma cells as well as 3T3 fibroblast cells and HT29 colon adenocarcinoma cells. Image series were segmented into cell-covered and cell-free areas. As patterning proceeds there is an increase in the size of cell-free areas (holes), and the rate of increase is characteristic for each cell type (Fig 1E). The patterning process is rapid for highly contractile cells (endothelial cells and fibroblasts exert 1000-2000 Pa traction stress [25, 37, 38]), and slow for weakly contractile colon carcinoma cells (which exert 200 Pa traction stress [39]).

**Fig 5:**
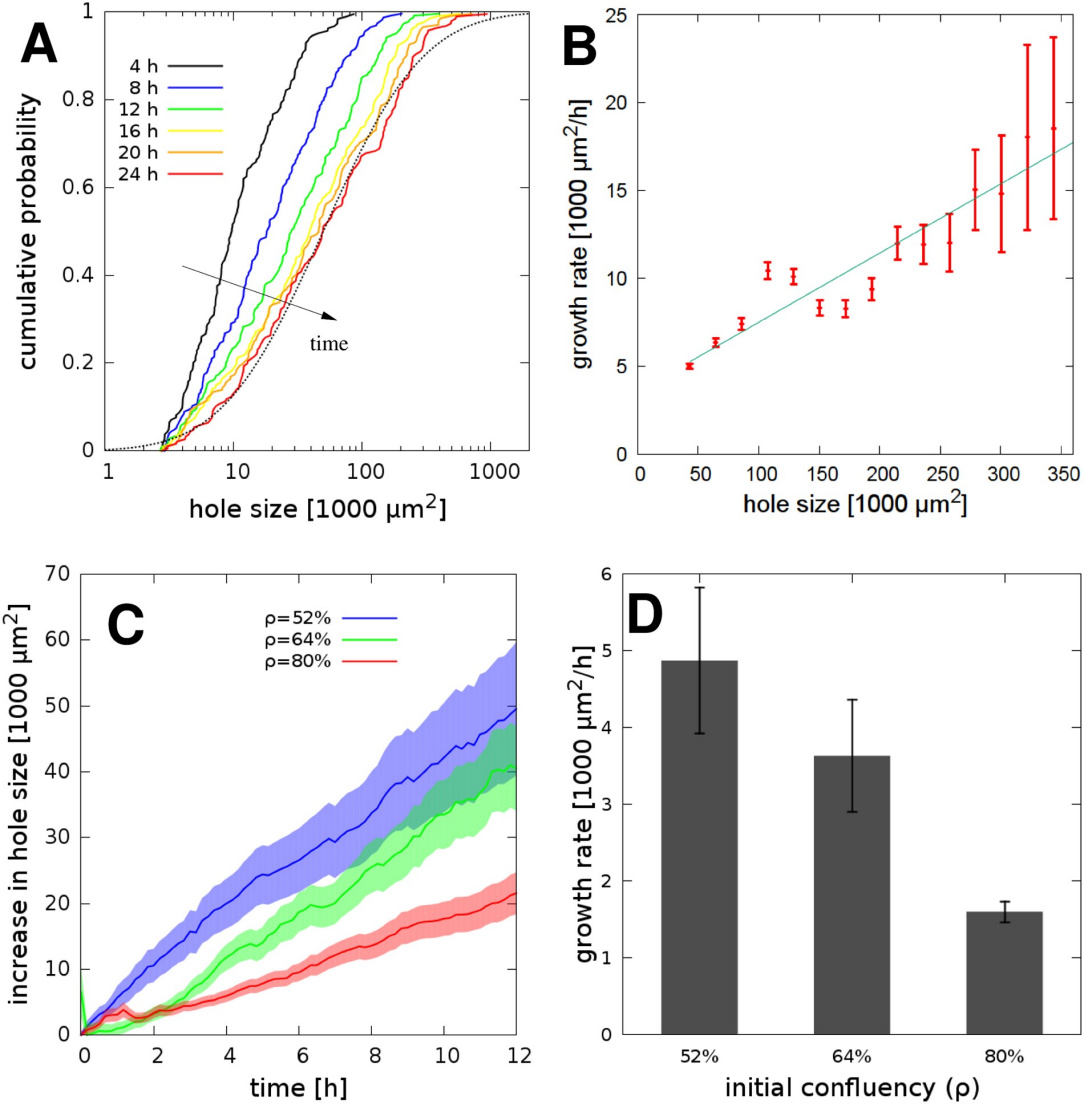
Quantitative evaluation of the gel patterning assay with A431 cells. A: Time-dependent cumulative distribution of cell-free area (hole) sizes: the fraction of holes that are smaller than the x-axis value. Initial cell coverage was 66%, distribution functions were compiled from *n* = 3 independent recordings and a total of 9 microscopic fields. The dotted line shows a lognormal distribution, serving as a guide to the eye. B: Expansion rate of cell-free areas, binned according to size. We identified areas that did not merge with adjacent holes during a 30 minute time interval, and determined the change in their area. Larger areas grow faster as the linear regression fit indicates (correlation coefficient: 0.94). Error bars indicate SEM. Data was pooled and binned from *n* = 3 independent sets of experiments. C: Comparison of patterning started at various initial cell densities. Time dependence of the mean hole size $\bar{A}$, compared to the initial value as $\bar{A}\left(t\right)-\bar{A}\left(t=0\right)$. Blue, green and red lines correspond to cultures seeded with initial cell coverage at 40%, 64% and 80% confluency, respectively. The growth rate of holes *r* decreases with increasing the initial cell density. Error stripes represent SEM, calculated from n = 3 independent sets of microscopic fields. D: Cell density-dependence of the hole size growth rate *r*, calculated from the curves depicted in panel C by linear fitting. Error bars indicate SEM.

In the following we focus our analysis on A431 epithelial carcinoma cells. This cell line was chosen due to its epithelial morphology and low two-dimensional motility when grown on tissue culture plastic surface—hence the patterning process is not compounded with substantial cell mixing within the monolayer. After segmenting the images into cell-covered and cell-free areas, we established the time-dependent distribution of cell-free areas (holes) as a measure to characterize the pattern (Fig 5A). Over the course of a day the mean pattern size increased ten-fold from 0.01 mm^2^ to 0.1 mm^2^. The coarsening process involves the expansion of individual cell-free areas as well as the merger of adjacent holes, and retain an approximately lognormal distribution of hole sizes—in good agreement with model predictions (Fig 3A).

As in model simulations (Fig 3B), larger holes grow faster (Fig 5B). The initial lag phase is often difficult to observe due to the time needed to set up the recording equipment after seeding the cells. The emergence of large enough holes is followed by a rapid expansion phase when the average hole size $\bar{A}$ increases with an approximately steady rate *r*:

$$\bar{A}\left(t\right)=\{\begin{array}{cc} r(t-\tau ) & \text{for}\;t\geq \tau \\ 0 & \text{for}\;t<\tau \text{.} \end{array}$$ (4)

Comparison of the approximate functional form (4) and the scaling relation (3) for *t* > *τ* yields

$$F^{*}∼r=\frac{d\bar{A}}{dt},$$ (5)

a convenient way to characterize cell contractility with the easily measurable rate of hole expansion *r* after the onset of patterning. The slower than exponential increase in $\bar{A}$ could reflect non-stationary experimental conditions: the contractility of cells decreases in older cultures as cells consume the growth factors and nutrients within the medium. Alternatively, an important difference between the model and real cells is volume exclusion: while in simulations particles can achieve an arbitrary high density, the volume of real cells would set a lower limit for equilibrium link lengths, and hence slow down the patterning process when this limit is approached and cells cannot contract further.

Our initial observations as well as model simulations indicate that the seeding cell density *ρ* is a sensitive parameter that determines the pace of patterning *r*. Thus, we performed experiments with three distinct seeding densities (Fig 5C). The overall expansion rate increased 3-fold when we compared a culture seeded at *ρ* = 80% confluency to one seeded at *ρ* = 50% confluency (Fig 5D). A linear ansatz

$$\frac{r}{r_{0}}=1-c\left(\rho -\rho_{0}\right)$$ (6)

is fitted to the data in Fig 5D, indicating that at *ρ*_0_ = 64% seeding confluency a 1% increase in cell density yields a c = 3.22% decrease in the hole size growth rate *r*. While local cell density is difficult to set precisely in the experiments, it is readily measurable after seeding. The calibration data in Fig 5C then offers the possibility to offset the effect of seeding density and derive standardized hole size growth rates *r*_0_, i.e., rates that are expected under the same experimental conditions at *ρ*_0_ = 64% seeding confluency. Using the linear ansatz (6), the standardized hole size growth rate *r*_0_ is given as

$$r_{0}=\frac{r}{1-c(\rho -\rho_{0})},$$ (7)

where *ρ* is the actual seeding density, *r* is the observed hole size growth rate and the normalization factor is *c* = 3.22%/%.

**Fig 6:**
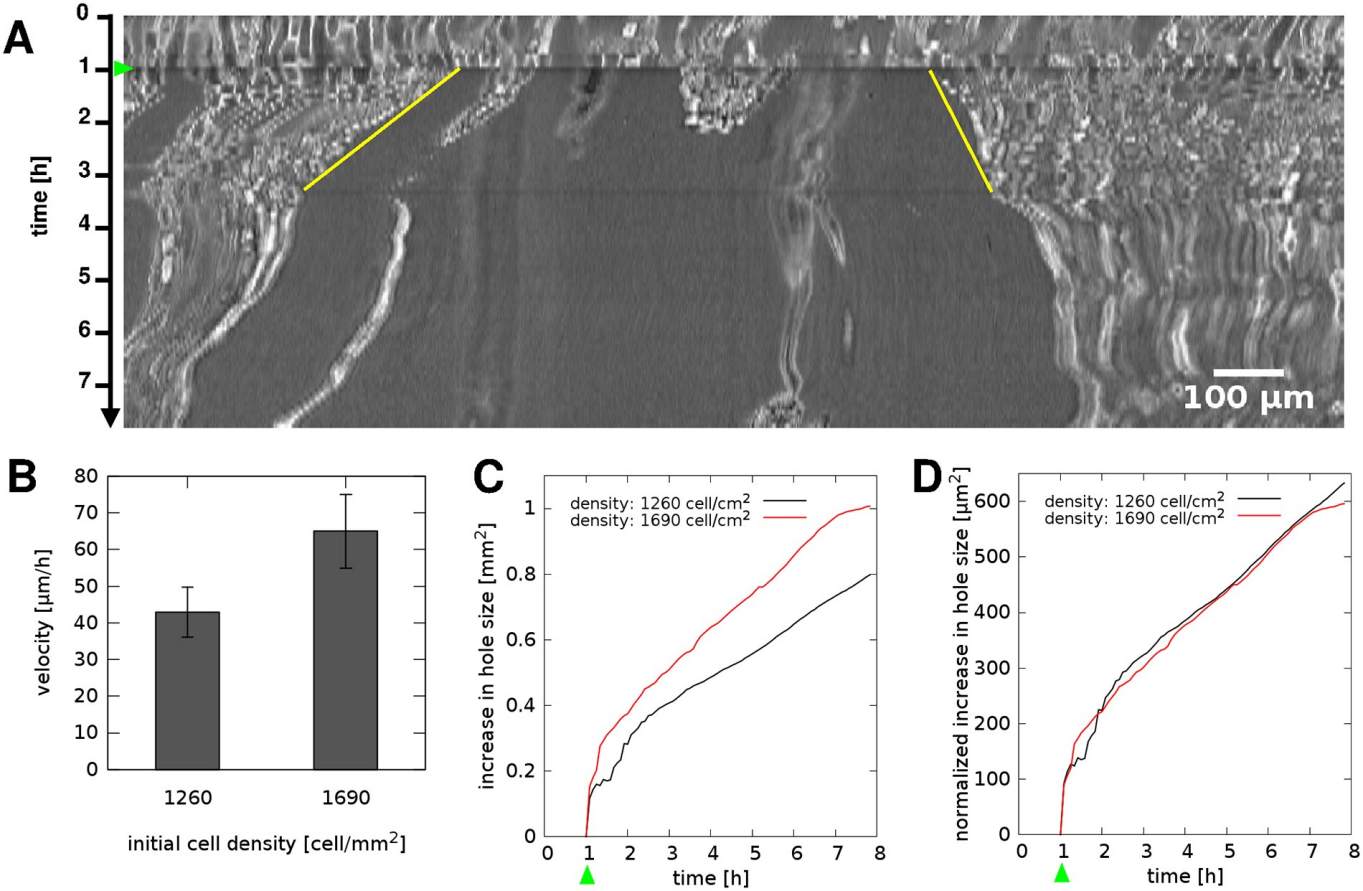
Tensile stresses within the cell-populated Matrigel expand a punctured wound. A: Typical kymogram depicting the expansion of a 0.2 mm^2^ punctured area (see also S8 Movie). A431-GFP cells were seeded at a density of 1690 cells/mm^2^, corresponding to 66% confluency. Green arrowhead indicates the time of the injury, yellow lines mark the boundary of the expanding wound during the subsequent 2 hours. Scale bar: 100 μm. B: Velocity of the expanding wound boundary, obtained from cultures seeded with two distinct cell densities. Error bars indicate SEM (n = 8). C: Cumulative change in wound area, determined by image segmentation, in two cultures seeded with distinct cell densities. Green arrowhead indicates the time of injury. D: Contribution of a single cell to the enlargement of the wound. Data shown in panel C were normalized with the corresponding initial cell densities.

We explain the Matrigel patterning assay by assuming that cells maintain a steady contractile state, which reorganizes the highly pliable ECM culture environment in a spatially inhomogeneous, non-linear manner. In particular, we assume that contractile stresses within the cell-ECM composite are proportional to the local cell density, and the speed of patterning is set by the creep rate of plastic deformations within the cell-ECM composite material. To verify this latter assumption, we evaluated patterning assays with more pliable, diluted Matrigel coatings. As expected, the patterning process was faster on diluted Matrigel (S1 Fig). To probe the presence of mechanical stresses directly, we performed mechanical puncture experiments. Such assays evaluate the initial movement around a suddenly imposed discontinuity. Expansion or shrinkage of the wound is expected to indicate the presence of tensile or compressile stresses within the material, respectively [40–42]. By using a plastic pipette tip we thus punctured the cell-Matrigel composite material, and recorded the wound area in every 5 minutes. Consistent with the presence of a contractile tension in the material, the wounds expanded immediately after the injury. We identified the movement of the boundary on kymograms and calculated the initial expansion speed of the wound. A comparison of cultures seeded with distinct cell densities indicates that the expansion speed is proportional to cell density and thus suggests that each cell is an active generator of tensile stress (Fig 6).

### NMII inhibitors perturb multicellular contractility

To further calibrate the Matrigel patterning process as an assay for cellular contractility, we used two inhibitors to interfere with NMII activity. Blebbistatin is a specific allosteric inhibitor of all type II myosins, including NMII [43]. Blebbistatin stabilizes type II myosins in the low-affinity actin binding conformation and also inhibits their ATPase activity. The compound Y27632 is a cell permeable inhibitor of Rho kinase (ROCK), which activates NMII by two distinct mechanisms [44]. ROCK directly activates NMII by phosphorylating the NMII regulatory light chain. An indirect activation involves the inhibition of the myosin light-chain phosphatase and thereby further shifting the equilibrium towards the active form of NMII. Both of these activation pathways are blocked by Y27632. The other activator of NMII, myosin light chain kinase, remains unaffected by Y27632.

**Fig 7:**
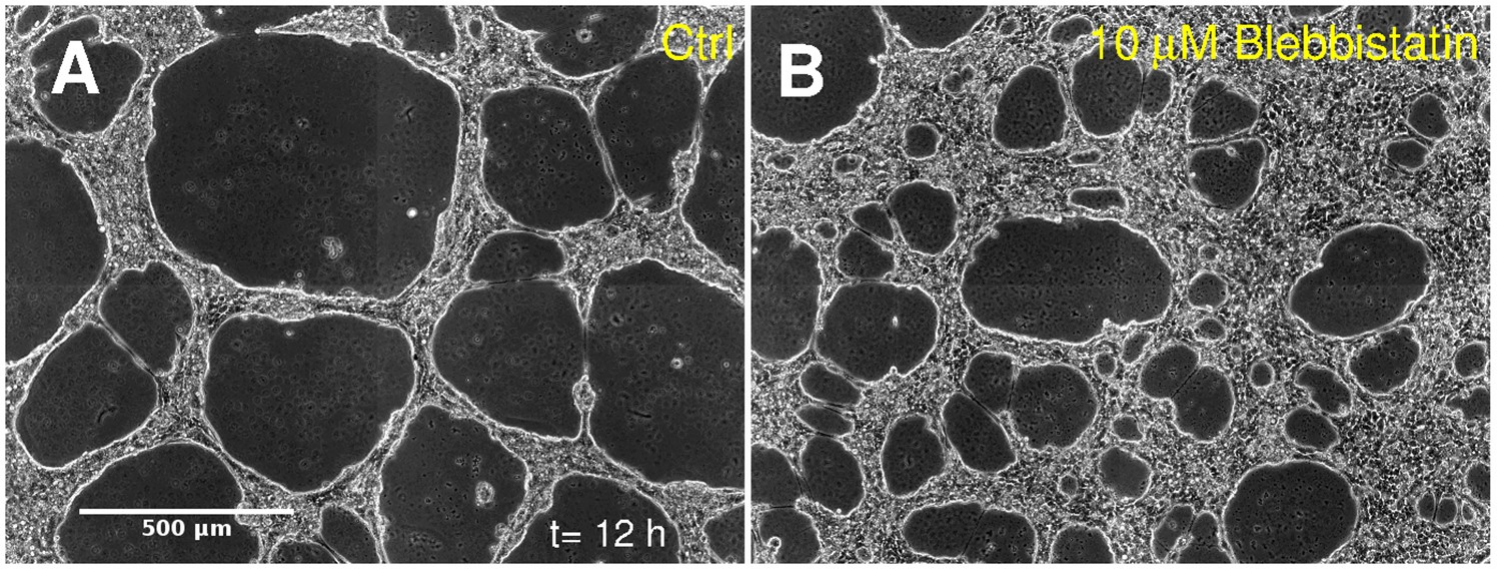
Non-muscle myosin II (NMII) is a key factor in multicellular contractility and pattern formation. Untreated A431-GFP cultures (A) exhibit larger structures than cultures in which NMII-based contractility was perturbed by 10 μM blebbistatin (B). Initial cell density was 1600 cells/mm^2^ (66% confluency) in all conditions, images shown were recorded after 12 h in culture (see also S9 Movie). Scale bar: 500 μm.

**Fig 8:**
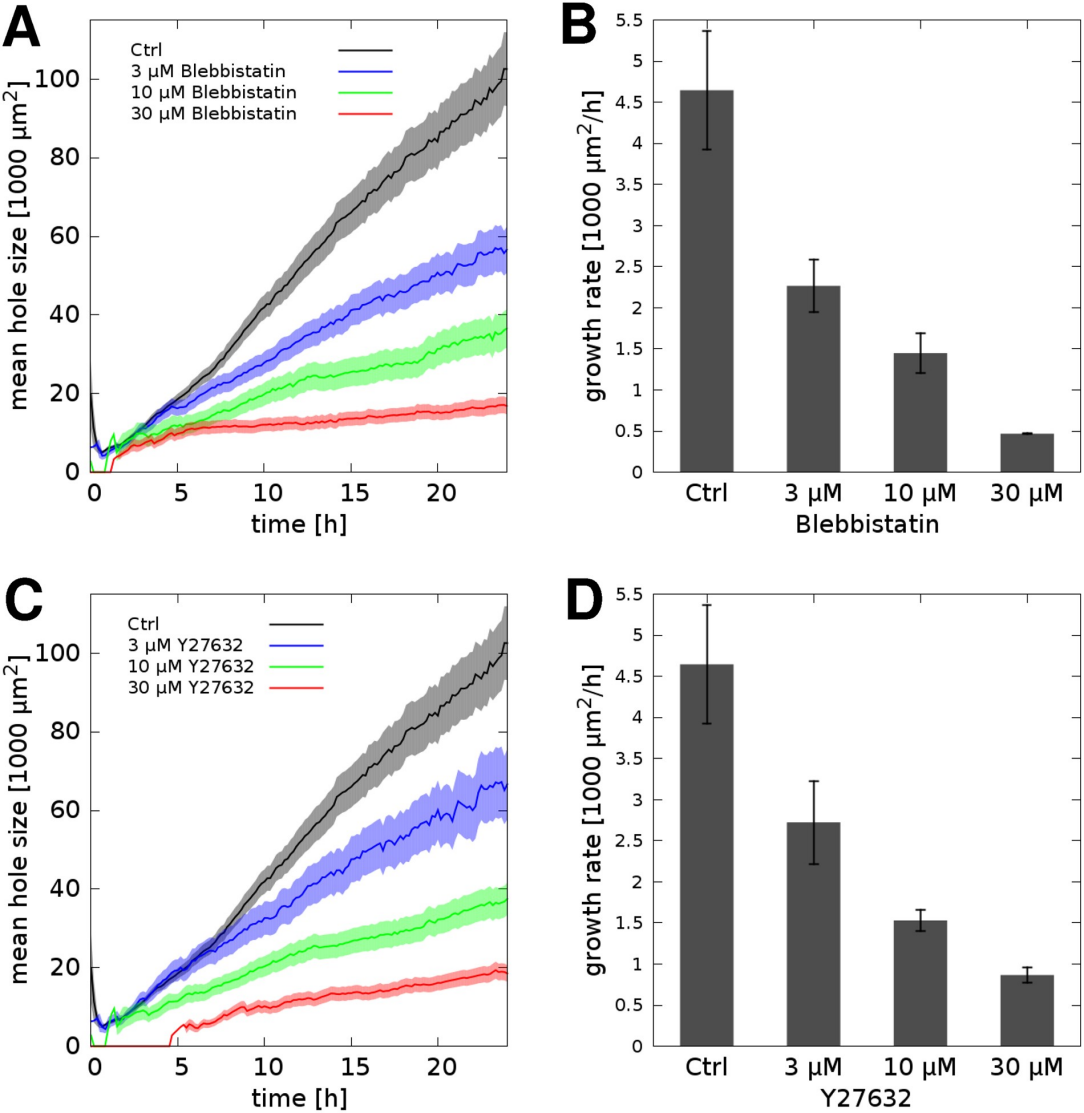
The patterning process slows down in the absence of normal NMII function. A, C: Time-dependent average hole sizes, $\bar{A}\left(t\right)$, indicate a concentration-dependent inhibition by blebbistatin (A) or by Y27632 Rho kinase inhibitor (C). B,D: Standardized hole expansion rates *r*_0_, extracted by linear fitting, characterize patterning speed and hence contractility. Cells were seeded at 66% confluency. Error stripes and bars correspond to SEM values, each obtained from data in n = 3 independent sets of microscopic fields.

As expected, dose-dependent inhibition of NMII activity by blebbistatin or Y27632 Rho kinase inhibitor yields a concentration-dependent reduction in the rate of hole expansion by A431-GFP cells, seeded at 66% confluency (Fig 7, S9 Movie). Accordingly, after 24 h in culture the cell-free areas were substantially smaller in NMII-inhibited cultures (Fig 8). In particular, areas larger than 1 mm^2^ were completely eliminated (S2 Fig). According to Figs 8 and 4, we estimate that 10 μM blebbistatin or 30 μM Y27632 reduces contractility of A431 cells by 3- and 6-fold, respectively.

### Role of S100A4 in multicellular contractility

Tensile cytoskeletal stresses arise when actin filaments, oriented in various directions, are stretched and pulled by NMII minifilaments. Multimerization of NMIIA, an isoform of NMII, can be regulated by binding of S100A4 protein [23]. S100A4 can bind and mask the assembly competence domain of NMIIA preventing its assembly into functional multimers or facilitating the disassembly of existing filaments, thereby controlling cell contractility. To study this fundamental aspect of NMII function, we overexpressed two S100A4 variants in A431 human epithelial carcinoma cells. Earlier works already established several aspects of the S100A4-myosin IIA interactions in A431 cells [45], offering a molecular context to the results of our functional assay.

**Fig 9:**
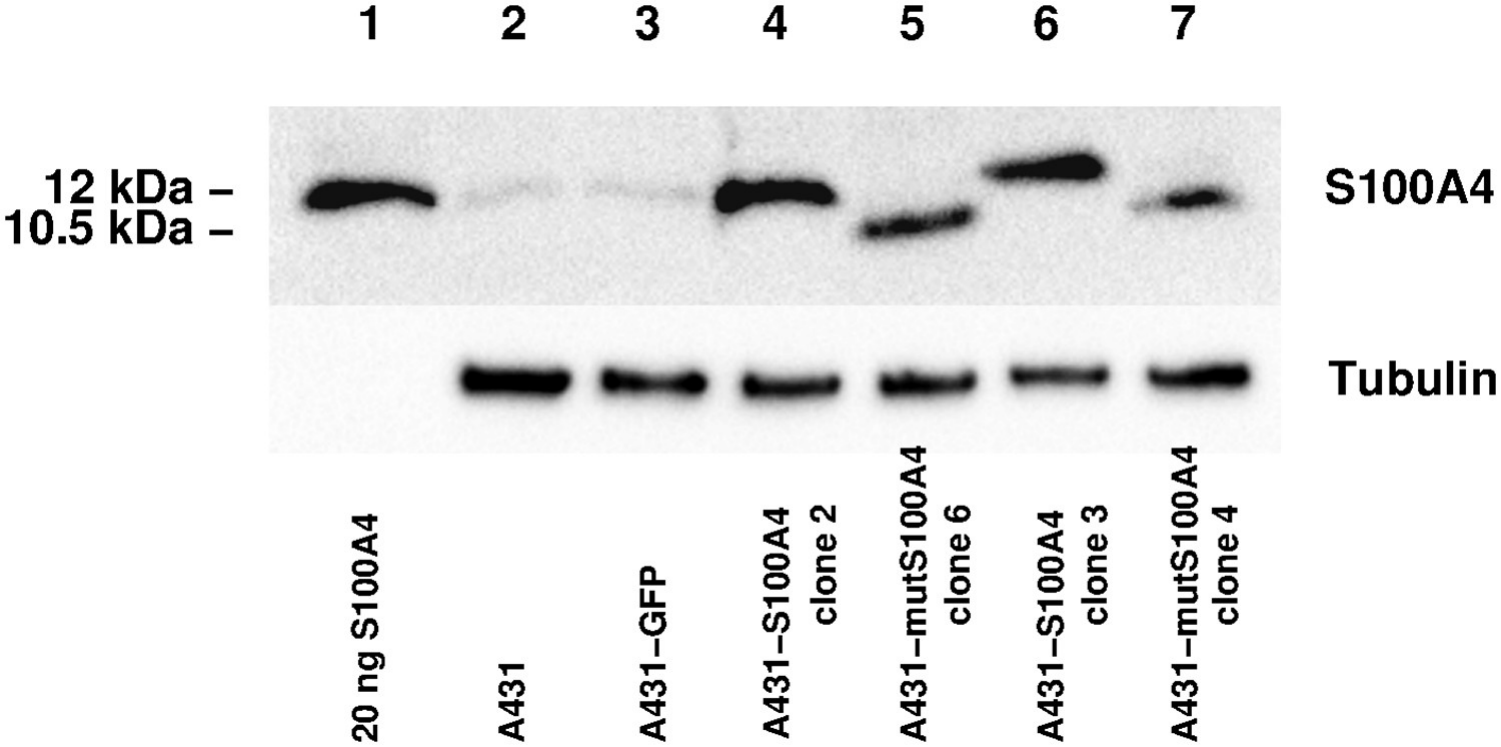
Western blot of S100A4 isoforms in A431 human epithelial carcinoma clones. Upper panel: S100A4 protein was detected in immunoblots of A431 clones. Lysates from A431 cells (lane 2), GFP-expressing A431 cells (lane 3) or clones overexpressing either wild type S100A4 (lanes 4 and 6), or truncated non-functional mutS100A4 (lanes 5 and 7) are shown with a standard recombinant wild type S100A4 protein sample for comparison (lane 1). Wild type S100A4 has a relative molecular mass of 12 kDa whereas truncated mutS100A4 is 10.5 kDa. Lower panel: For loading control, the upper part of the blot was immunolabeled for *β*-tubulin (50 kDa).

Wild type S100A4 is capable of binding to the assembly competence domain of NMIIA and thus prevents the assembly of NMIIA dimers and tetramers. The mutant S100A4 isoform (mutS100A4) does not prevent NMIIA filament assembly as it contains a point mutation (Cys81Ser) in the hydrophobic binding pocket responsible for binding to NMIIA. Additionally, mutS100A4 lacks 13 C-terminal amino acid residues, which further decreases its affinity to binding partners. Thus, as negative controls in S100A4 functional assays we used A431 clones stably transfected with the mutS100A4 construct, a clone transfected with GFP only as well as non-transfected A431 cells. Western blots indicate stable expression of both the wild type and mutant S100A4 variants in A431 clones (Fig 9).

**Fig 10:**
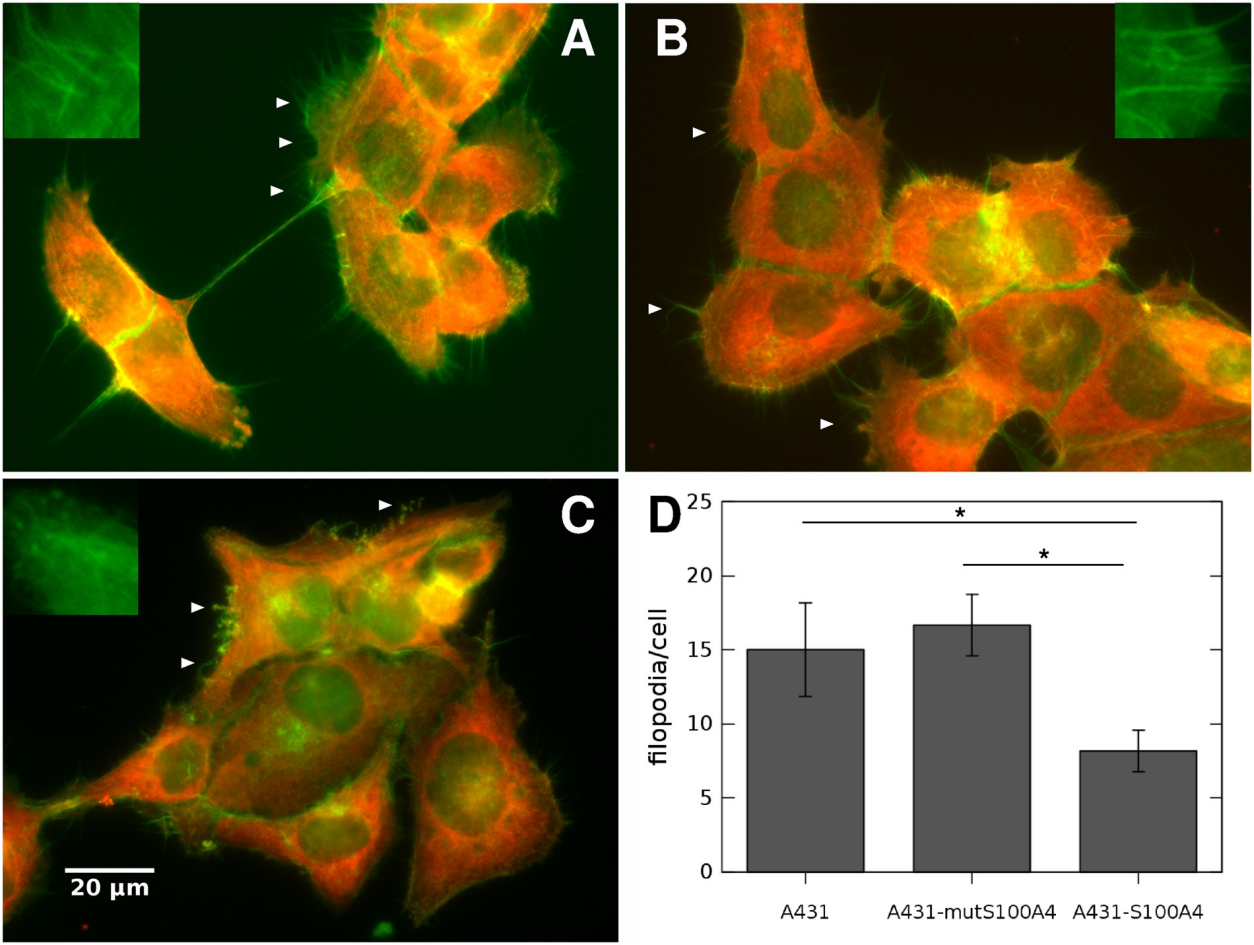
The cytoskeleton of A431 clones overexpressing wild type or mutant S100A4. Normal A431 cells (A) and A431 clones overexpressing either non-functional mutS100A4 (B) or wild type S100A4 (C) were immunolabeled for NMIIA (red) while actin filaments were visualized by fluorescein-labeled phalloidin (green). NMIIA is ubiquitous in each clone, while actin filaments and filopodia are less abundant in the clone expressing wild type S100A4 (C). White arrowheads point to filopodia. Scale bar: 20 μm, 40X objective. Insets in A-C show characteristic F-actin structures within 10 μm wide areas. D) Filopodia count, averaged from *n* = 6 representative cells for each group. Error bars represent SEM and asterisks indicate statistically significant difference with *p* < 0.05.

Western blots from cell lysates were also used to verify that the myosin variant NMIIA is indeed present in A431 human epithelial carcinoma cells (S3 Fig). Double labeling of NMIIA and F-actin in normal A431 cells shows that NMIIA is present in cytoplasmic speckles with structures that tend to colocalize with F-actin where actin stress filaments are present (S4 Fig). NMIIA remains ubiquitously present in the cytoplasm of A431 clones overexpressing different S100A4 variants (Fig 10). However, the overexpression of wild type S100A4 alters the organization of the cytoskeleton. The most conspicuous difference is in filopodium density: F-actin rich filopodia are abundant in both normal A431 cells and in the clone expressing the non-functional mutS100A4 protein, whereas filopodia become shorter and fewer with less stress filaments when wild type S100A4 is overexpressed.

**Fig 11:**
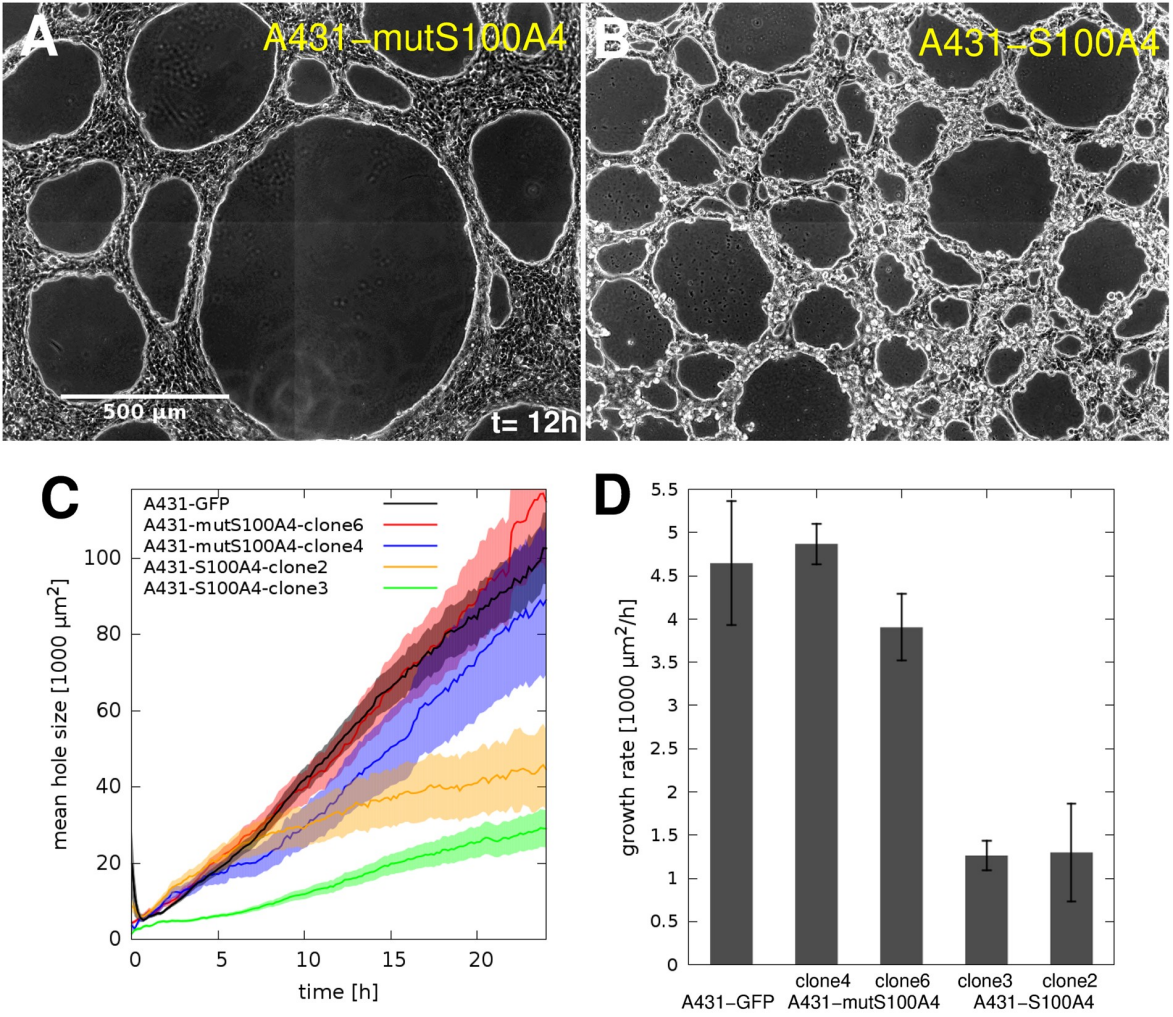
Effect of S100A4 protein on Matrigel patterning of A431 cells. A-B: representative images taken at 12 h in culture (see S10 Movie) of A431 cells overexpressing either the non-functional mutant form (A431-mutS100A4, clone6) or the wild type S100A4 (A431-S100A4, clone2). Initial cell density was 1600 cells/mm^2^ (66% confluency). Scale bar: 500 μm. C: Time-dependent average hole sizes, $\bar{A}\left(t\right)$, obtained for 5 distinct A431 clones. D: Standard hole size growth rates *r*_0_ were extracted from the data shown in C by linear fitting. Error stripes and bars are SEM values, each calculated from n = 4 independent experiments.

To test the effect of S100A4 on multicellular contractility-driven patterning of A431 cells we employed the clones overexpressing either wild type S100A4 or the non-functional mutant isoform of S100A4 as well as a GFP-expressing clone for comparison. When seeded on Matrigel, the overexpressed wild type S100A4 reduced the rate of hole expansion by ≈75% (Fig 11). Accordingly, after 15 hours the size distribution of cell free areas indicates the absence of large holes (S5 Fig). Comparing these effects with the experiments utilizing small molecule inhibitors of NMII (Fig 8), the overexpression of S100A4 reduces cell contractility to an extent comparable with blebbistatin or Y27632 at 10 μM concentrations. Based on our numerical model, the observed behavior is consistent with a 75% reduction in cell contractility.

## Discussion

### Contractility assay

To study NMII-dependent multicellular contractility, we developed and calibrated a bioassay utilizing the ability of contractile cells to pattern a soft Matrigel substrate into network structures. The imaging-based assay was interpreted by a computational model that keeps track of intercellular connections and allows the relaxation of mechanical stresses by rearranging the connectivity of particles. The model does not distinguish between the cell and ECM component of the composite material: as both components move together and form a composite material, model particles represent both a cell and a surrounding ECM microenvironment. While our model incorporates the essential biomechanical aspects of the patterning process, simplification during the modeling approach—like assuming an environment in steady state and not considering volume exclusion effects—limits the quantitative applicability of our model to the onset of pattern formation.

In our experiments we utilized Matrigel [46], a uniquely pliable biological hydrogel. The 200 Pa yield stress of Matrigel [47] is thirty times lower than the 6 kPa yield stress of a type I collegen gel at 2 mg/ml concentration [48]. In comparison, typical traction stresses are 1500 Pa for endothelial cells [37], 400-2000 Pa for fibroblasts [25, 38], 1000 Pa for A431 epithelial carcinoma cells [49] and 200 Pa for colon carcinoma cells [39]. Thus, the yield stress of Matrigel is well within the range of cell-exerted stresses, while the yield stress of collagen I is not. Accordingly, we did not expect to observe and were unable to elicit similarly quick pattern formation on collagen I or fibrin gel substrates. Cell contractility-driven patterning of collagen gels takes several days to weeks and yield different patterns [30, 31] with an important contribution of cell motility [32–35].

The unique mechanical properties of Matrigel could be mimicked by the synthetic polymer hydrogel PMEDSAH (poly[2-(methacryloyloxy)ethyl dimethyl-(3-sulfopropyl)ammonium hydroxide]). PMEDSAH is a suitable substrate for cell adhesion [50], and exhibits an elastic modulus of 380 Pa [51], a value similar to 450 Pa, the elastic modulus of Matrigel [52]. The yield stress of PMEDSAH is likely to be higher than that of Matrigel. The best estimate for the yield stress of PMEDSAH comes from a recent report [53] where a yield stress of 1500 Pa was measured for a composite material of 75% PMEDSAH and 25% pellethan fibermat. Based on this report the yield stress of PMEDSAH is expected to fall in the upper range of cell-exerted stresses. Therefore, PMEDSAH is a promising synthetic substrate candidate to measure multicellular contractility.

While the patterning assay does not allow absolute determination of cell-exerted forces, it readily provides relative comparison between parallel cultures after differences in initial seeding density are properly factored into the evaluation. Importantly, we found that A431 epithelial carcinoma cells in the gel patterning assay responded to inhibitors of NMII activity at concentrations that overlapped with the range used previously in cell traction force microscopy studies (50 μM for blebbistatin and 10-25 μM for Y27632 [54–57]). All internal control (untreated) cultures in *n* > 10 experiments, performed over the time span of several years, yielded consistent numerical values for the standardized patterning speed *r*_0_. This consistency allows to measure dose-dependent responses in parallel cultures and thus allows future development of medium- to high-throughput applications to evaluate the effects of pharmacological compounds on cell contractility.

### Endothelial tube formation assay

Multicellular contractility-based pattern formation is not limited to epithelial or endothelial cells that exhibited very similar dynamics in our experiments. Various cell types including fibroblasts and smooth muscle cells have been reported to generate network patterns when cultured on sufficiently malleable substrates [58]. Still, the endothelial tube formation assay on Matrigel substrate [59, 60] is widely used as a functional assay not only for endothelial cells but also for tumor cells exhibiting vasculogenic mimicry [61–63]. Currently a wide range of commercially available angiogenesis assays employ cells seeded on Matrigel or other basal membrane extract substrates, and angiogenic tube formation potential is evaluated by characterizing the emerging network pattern.

We, however, argue that these assays are primarily sensitive to multicellular contraction, and the characteristic network patterns are distinct from—and formed by a different mechanism than—lumenized endothelial tubes [64]. The fundamental difference in the patterning mechanism of the Matrigel assay and of angiogenesis is also reflected in the computational models describing the two phenomena. The primary process of angiogenesis is multicellular sprouting, which likely involves chemotactic guidance [65–69], contact guidance by ECM structure [70–72], or by cell-cell contacts [73, 74]. In contrast, cells in the Matrigel patterning assay are repositioned by a contractility-driven convective plastic flow. Thus, appropriate care should be taken when interpreting tube formation assay results, especially taking into consideration the ubiquitous ability of contractile cells to form network-like structures by deforming the elastoplastic cell-ECM composite material.

### Biomedical role of S100A4-mediated multicellular contractility

Decreased contractility in vivo can lead to altered tissue integrity such as in various tumors and it can be a step towards a metastatic phenotype. Increased levels of S100A4 were reported for several tumors [75–77] and it also correlates with worse prognosis in cancer patients [78]. By binding to extracellular partners like annexin A2 and transglutaminase-2 [79, 80], S100A4 can also hinder cell-ECM adhesion of cancer cells [81]. The observed change in stress filament abundance (Fig 10) is in agreement with findings in MDA-MB-231 breast carcinoma cells, which exhibited an increase in cytoskeletal stress filament formation in the absence of S100A4 [82]. Although several aspects of the relationship of S100A4 with NMII and the cytoskeleton have been elucidated, the direct impact of S100A4 on cell contractility using a functional assay has not yet been reported to our knowledge. In this paper we have demonstrated that S100A4 expression can indeed modulate tissue-level contractility of epithelial carcinoma cells in vitro.

### Conclusions and outlook

In this work we utilized cell-resolved simulations, but we expect that the same mechanism can be also captured by a fluid dynamics approach [83–85]. We trust that the presented bioassay will be helpful as a relatively simple tool to further elucidate the roles of cell contractility and its molecular regulators. We identified key experimental variables such as cell density and ECM pliability which must be tightly controlled in experiments or taken into account when analyzing results. While we used a time-lapse approach, hole size distribution functions obtained from end-point micrographs are also likely to reveal contracile activity (see S2 and S5 Figs).

## Methods and models

### Cell culture

The human A431 epithelial carcinoma cell line, 3T3 mouse fibroblast cell line and HT29 human colon carcinoma cell line were obtained from ATCC. Cells were maintained in Dulbecco’s Modified Eagle Medium (DMEM, Lonza) containing L-glutamine, supplemented with 10% Fetal Bovine Serum (Invitrogen) and Penicillin-Streptomycin-Amphotericin B (Lonza). Primary human umbilical vein endothelial cells (HUVEC) and primary human cardiac microvascular endothelial cells (HMVEC-C) were purchased from Lonza and cultured in EGM2 medium (Lonza) containing Penicillin-Streptomycin-Amphotericin B (Lonza).

### S100A4 constructs

The gene of human S100A4 (Uniprot code: P26447) was obtained from Dr. Jörg Klingelhöfer. We derived a mutant isoform, herein referred to as mutS100A4, containing a point mutation in position 81 that replaces a cysteine by serine and lacking 13 amino acids at the C-terminal, by the Megaprimer method [86]. Both the wild type S100A4 and the mutS100A4 genes were subcloned into pIRES2-eGFP plasmid (Clontech), containing an internal ribosome entry site using restriction sites XhoI and BamHI. Cells were transfected with linearized plasmids (using BsaI restriction site for linearization) using FuGene HD transfection reagent (Promega), according to the manufacturer’s instructions. Stable transfectants were selected with 0.4 mg/ml G418 antibiotics (Merck Millipore). After two weeks of selection, stably transfected cells were further selected by their GFP signal using FACSAria Cell Sorter (BD Biosciences). After selection, cells were maintained in 0.2 mg/ml G418. Six S100A4-overexpressing and five mutS100A4-overexpressing cell clones were eventually established, of which two from each group were used for detailed studies.

### Recombinant proteins

The gene of human S100A4 was cloned into a modified pBH4 expression vector, expressed and purified as described earlier [21].

### Inhibitors

Y27632, the cell permeable inhibitor of Rho kinase was purchased from Merck Millipore, dissolved in water to make 10 mM stock solutions and used in final concentrations up to 30 μM. Blebbistatin, the inhibitor of non-muscle myosin II, was purchased from Merck Millipore, dissolved in DMSO to make 50 mM stock solutions and used in final concentrations up to 30 μM, as indicated in the corresponding text. For negative control treatments identical amount of water or DMSO was used.

### Immunocytochemistry

To detect the NMIIA isoform in A431 cell line, cells were seeded into glass coverslip-containing 24-well plates. Cells were fixed with 4% paraformaldehyde and permeabilized using 0.1% Triton X-100. Samples were blocked with 2% w/v Bovine Serum Albumine solution, endogenous NMIIA was detected using anti-NMIIA antibody (rabbit, polyclonal, 1:300, Biolegend) and Alexa Fluor-546-conjugated anti-rabbit secondary antibody (Life Technologies). Actin filaments were stained by phalloidin-FITC conjugate (Sigma-Aldrich). Immunofluorescence was imaged using a Zeiss Axio Observer Z1 microscope equipped with 40x EC Plan-Neofluar objective, Colibri illumination system and AxioCam MRm camera.

### Western blot

A431 stable transfectants were lysed in lysis buffer (25 mM Tris pH 7.4, 150 mM NaCl, 2 mM EDTA, 1% v/v Triton X-100, 2.5 mM DTT and 1% v/v protease inhibitor cocktail). Protein concentration was measured by Bradford method, and 20 μg total protein samples were run by SDS-PAGE using 10% Tris-Tricine gel. Samples were blotted to PVDF membrane and S100A4 was detected by anti-S100A4 antibody (mouse, monoclonal, PR006.21.3, 1:1000 dilution, a kind gift of Dr. Jörg Klingelhöfer) and horseradish peroxidase-conjugated anti-mouse secondary antibody (1:5000, Santa Cruz). For loading control, tubulin was detected by anti-tubulin monoclonal antibody, (1:5000, Sigma) and horseradish peroxidase-conjugated anti-mouse antibody. Chemiluminescence was detected by using ECL Western Blotting Substrate (Pierce). NMIIA was detected from 20 μg A431 cell lysate using 8% Tris-glycine gel and anti-NMIIA antibody (1:2000, BioLegend) and horseradish peroxidase-conjugated anti-rabbit secondary antibody (1:2000, Santa Cruz).

### Live cell imaging

Time-lapse recordings were performed on a Zeiss Axio Observer Z1 inverted microscope with 10x Plan Neofluar objective. The microscope was equipped with a Zeiss AxioCam MRm CCD camera and a Marzhauser SCAN-IM powered stage. Cultures within tissue culture Petri dishes (Greiner) were kept in a stage-mounted incubator providing 37°C and a humidified 5% CO_2_ atmosphere. Stage positioning, focusing, image collection and stitching of images into mosaics (2x2, 3x3 or 9x12) were controlled by Zeiss Axiovision 4.8 software and a custom experiment manager software module. Phase contrast images were collected every 10 minutes from each microscopic field for durations ranging from 24 to 48 hours.

### Matrigel patterning assay

Matrigel substrates were prepared by pouring 20 μl ice cold Matrigel solution (ECM gel, E1270, Sigma) into 6 mm diameter circular wells. Three of such polylactic acid (PLA) well walls were previously filament-deposition (“3D”) printed (Ultimaker) into tissue culture Petri dishes (Greiner) using the method described earlier by our group [87]. After gelation, cell suspension was added to the wells at an average density of 1600 cells/mm^2^. Following an initial adhesion phase, cells covered approximately 2/3 of the Matrigel surface. Cells remodeled the Matrigel within a few hours, and the process was recorded by phase contrast time-lapse microscopy.

### Image analysis

Images recorded by time-lapse microscopy were analyzed using the NIH ImageJ software with plugins of our design, available at https://github.com/gulyasmarton/ContractilityAnalyzer. In the Matrigel contraction assay cell-free areas (holes) develop within the cell layer that was seeded on a Matrigel substrate. The images were filtered by a Gaussian blur (the width was adjusted to the sample) and were segmented by the default ImageJ thresholder (IsoData [88]). We identified each hole as a cluster of connected pixels and determined their areas. From this data we calculated the mean hole size and the cumulative distribution function of the hole areas as a function of time. We also determined the expansion rate of holes by identifying overlapping clusters on consecutive images. Hole merging and splitting events were excluded by selecting clusters that overlap with only a single cluster in the second image.

### Computational model describing an elastoplastic multicellular mechanics

**Fig 12:**
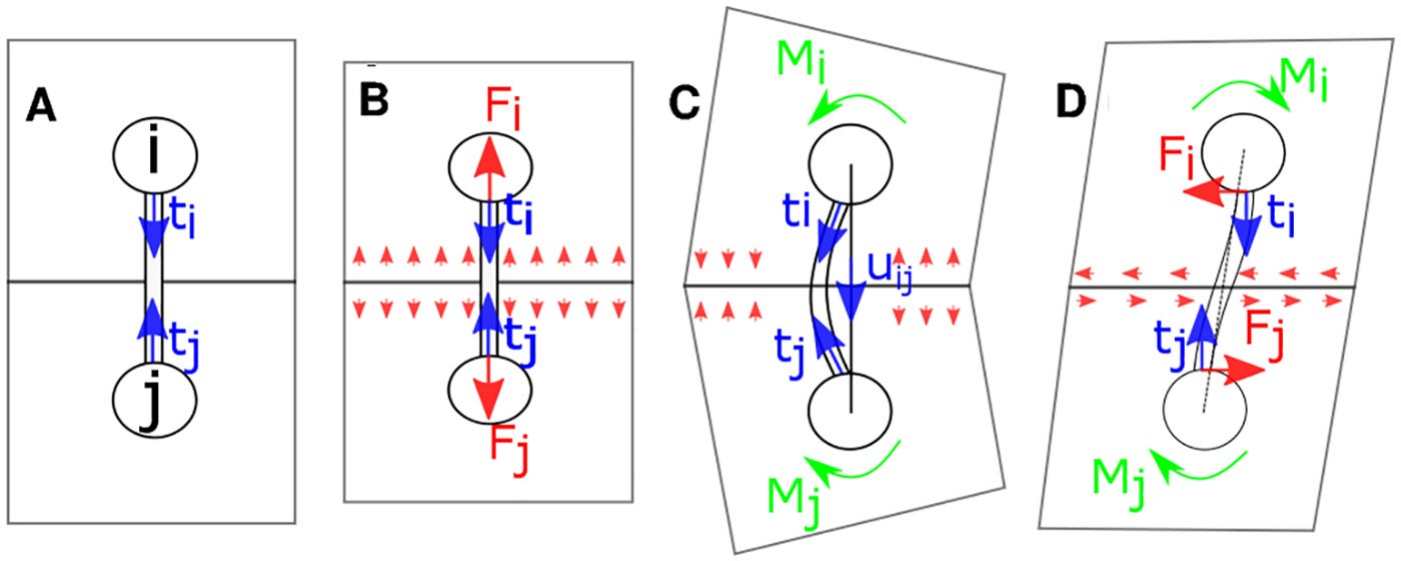
Mechanical model. Rectangular shapes indicate cell membranes. Beams represent mechanically connected cytoskeletal structures of adherent cells. A: Mechanical stress-free configuration of two particles, *i* and *j*. Unit vectors, **t**_*i*_ and **t**_*j*_ co-rotate with the particles. B: Compressed cells. Interaction of the two cells raise spatially distributed forces (red arrows), replaced by the repulsive net forces **F**_*i*_ and **F**_*j*_. C: Symmetric rotation of both particles yields torques **M**_*i*_ and **M**_*j*_ acting on particles *i* and *j*. Torque vectors are perpendicular to the plane of the figure. D: Shear of the tissue creates torques **M**_*i*_, **M**_*j*_ and also shear forces **F**_*i*_, **F**_*j*_ acting at the particles.

We used a computational model [36] that represents the elastoplastic mechanics of multicellular assemblies by a network of particles and interconnecting elastic beams (Fig 12), available at https://github.com/donnagreta/cellmech. Briefly, each particle *i* is characterized by its position **r**_*i*_ and orientation *ϕ*_*i*_. Since we consider a two dimensional system on the *x*-*y* plane, *ϕ*_*i*_ has a single component, describing rotation around the orthogonal (*z*) direction. The beams transmit torques and non-central forces (i.e., forces with shear components) that arise due to the relative movement of adjacent cells.

Hooke’s law determines the force *F*^∥^ that arises when a pair of cells is stretched or compressed (Fig 12b). For a link *l* interconnecting particles *i* and *j*, the Hooke force component is given as

$$\begin{array}{c} F_{l}^{∥}=k(|r_{j}-r_{i}\left|-\ell_{l}\right), \end{array}$$ (8)

where *ℓ*_*l*_ is the mechanically neutral distance of the two cells and *k* > 0 is a model parameter representing cytoskeletal stiffness.

A torque and shear forces are exerted by a link if its mechanically neutral (“preferred”) direction at particle *i*, **t**_*i*,*l*_, is distinct from its current direction **u**_*i*,*j*_ (Fig 12c and 12d). For small differences the exerted torque is proportional to the angle between the preferred and actual directions:

$$M_{i,l}=g\left(t_{i,l}\times u_{i,j}\right),$$ (9)

where the microscopic bending rigidity *g* is a model parameter setting the macroscopic shear modulus [36]. Shear forces transmitted by the link are calculated from the requirement of the link being in mechanical equilibrium.

Multicellular plasticity can be modeled with rules rearranging the network of intercellular adhesions. The probability of removing a link *l* during a short time interval Δ*t* increases with the tensile Hookean force (8) transferred by the link, $F_{l}^{t}$, as defined by Bell’s rule [89]:

$$p_{l}\Delta t=Ae^{F_{l}^{t}/F_{0}}\Delta t,$$ (10)

where *F*_0_ is a threshold value and *A* is a scaling factor defining stability of connections.

Mechanical connections can be established between two Voronoi neighbor particles, *i* and *j*. During a short time interval Δ*t* the probability of inserting a new link is a decreasing function of the distance *d*_*i*,*j*_ between the particles:

$$q_{i,j}\Delta t=B(1-\frac{d_{i,j}}{d_{\text{m}ax}})\Delta t,$$ (11)

where the scaling factor *B* defines the intensity of cellular protrusive activity and *d*_m*ax*_ denotes the maximal distance for new connections.

Using the probability distributions (10) and (11), the next event *μ* and waiting time *τ* is calculated according to the stochastic Gillespie algorithm [90]. The waiting time until the next event is chosen from the distribution

$$\text{log}\;P\left(\tau \right)=-\tau (\sum_{l}p_{l}+\sum_{i,j}q_{i,j}),$$ (12)

where the sums are calculated by iterating over each link *l* and over all possible Voronoi neighbor particle pairs *i*, *j* that are not connected by a link.

### Computational model of contractile cells on a Matrigel substrate

To interpret the results of the ECM contractility assay, we augmented the above model with rules that specifically control the equilibrium distance between adjacent cells and represent adhesion between cells and the culture substrate (Fig 2A).

Assuming that cells actively maintain a specific contractile environment, we set the equilibrium link length *ℓ*_*l*_ in Eq (8) as

$$\frac{d\ell_{l}}{dt}=Cd_{0}\frac{F_{l}^{t}-F^{*}}{F_{0}}.$$ (13)

Thus, the change in the equilibrium link length is proportional to the difference between a target contractility value, F*, and $F_{l}^{t}$, the tensile component of the actual Hookean force (8). Parameter *C* sets the temporal scale of the regulation. In expression (13) link lengths are compared to the average cell size, *d*_0_ ≈ 25 μm, and contractile forces are compared to the Bell threshold force *F*_0_.

Adhesion between the cells and the rigid substrate is mediated trough the elastoplastic Matrigel. The elastic component of the adhesion force is proportional to the displacement relative to an equilibrium position $r_{i}^{0}$ as

$$F_{i,0}=g_{0}\left(r_{i}-r_{i}^{0}\right).$$ (14)

Over a longer time scale the Matrigel dissipates elastic stresses by adjusting the equilibrium position as

$$\frac{dr_{i}^{0}}{dt}=\frac{\alpha d_{0}}{F_{0}}F_{i,0},$$ (15)

where parameter *α* sets the speed of Matrigel creep between the rigid substrate and the cell. The suite of simulation tools is accessible at https://github.com/aczirok/cellmech-contractilityAssay.

### Model parameters

The unit distance of the simulations was set to the average size of an A431 cell adhering to a rigid substrate, *d*_0_ = 25 μm. (On the soft Matrigel, however, cells are smaller and the corresponding cell density of 1600 cells/mm^2^ yields only 2/3 initial confluence.) The maximal distance, *d*_m*ax*_, that still allows two cells to establish mechanical contact by extending protrusions was chosen as *d*_m*ax*_ = 50 μm.

Our force unit—and the typical magnitude of the forces acting in the simulation—is the threshold force *F*_0_ ≈ 100 nN [36] which is needed to separate two adherent cells according to Eq (10). For elastic parameters we used values calibrated in [36]. A431 cells, like metastatic epithelial carcinoma cells, were assumed to be soft [91], thus for the elastic parameters of the cell-ECM composite we used *E* = 500 Pa, and the Poisson number *ν* = 0.1. These values translate to dimensionless microscopic parameters *k* = 1.5 and *g* = 1. We set the elastic modulus associated with substrate adhesion to the same magnitude: *g*_0_ = 1.5.

Waiting times between simulation events are set by parameters *A* and *B*. We choose our time unit as 1/*B* ≈ 1 min, the time needed for two adjacent cells to build up molecular complexes that mechanically link their cytoskeletons. We set the lifetime of an unloaded link to 1/*A* ≈ 3 h. Thus, according to these values, two cells pulled away by the threshold force *F*_0_ separate in ≈1 h, a value consistent with the time scale observed in our cell culture experiments (Fig 5). Similar time scales were set for contractility regulation (1/*C* ≈ 20 min), and for the speed of Matrigel creep *αd*_0_/*F*_0_ = 1.5 μm/h / nN. According to the latter choice, the force unit *F*_0_ induces a creep flow at the speed of ≈ 150 μm/h—a value consistent with the observed speeds in our culture system. While we report simulation results with these parameters, parameter sensitivity analysis indicates that none of these values are crucial to obtain the reported patterns.

## Supporting information

S1 FigEffect of Matrigel dilution on patterning.Time dependence of the mean hole size (cell-free area), $\bar{A}$) of the developing pattern. A431-GFP cells were seeded at 1600 cells/mm^2^ initial density on Matrigel or Matrigel diluted to 90% v/v with PBS. Error stripes represent SEM, calculated from n = 3 independent sets of experiments.(TIF)

S2 Fig Inhibition of NMII function. Either blebbistatin (A) or Y27632 Rho kinase inhibitor (B) effectively blocks the formation of large holes in a concentration dependent manner within the Matrigel patterning assay. Cumulative distribution functions of particle free area (hole) sizes indicate the fraction of holes that are larger than the value at the abscissa. Each distribution was pooled from n = 3 sets of microscopic fields, imaged 15 h after seeding A431 epithelial carcinoma cells on Matrigel substrate.
(TIF)

S3 Fig NMIIA western blot of A431 human epithelial carcinoma cell clones. Upper panel: Lysates of A431 cells (lane 1), GFP-expressing A431 cells (lane 2) and clones overexpressing either wild type S100A4 (lanes 3 and 5), or truncated non-functional mutS100A4 (lanes 4 and 6) were immunoblotted for non-muscle myosin II A isoform. NMIIA is present in all clones with a relative molecular mass of around 200 kDa. Lower panel: For loading control, the lower part of the blot was immunolabeled for *β*-tubulin (50 kDa).
(TIF)

S4 Fig Colocalization of F-actin and NMIIA in A431 cells. F-actin was visualized by fluorescein-labeled phalloidin (panel A, green in panel B), while NMIIA was immunolabeled (panel C, red in panel B). Colocalization is seen as structures of yellow color (panel B) and pointed to by yellow arrowheads in the insets of panels A and C. Scale bar: 20 μm, 40X objective.
(TIF)

S5 Fig Effect of S100A4 on the contractility of A431 cells, characterized by the distribution of hole sizes. Cumulative distribution functions of particle free area (hole) sizes indicate the fraction of holes that are larger than the value at the abscissa. Each data set was pooled from n = 4 independent sets of microscopic fields, imaged 15 h after seeding A431 epithelial carcinoma cells on Matrigel substrate. A431 clones were either overexpressing a non-functional mutant S100A4 (clone6: red and clone4: blue), the wild type S100A4 (clone2: orange and clone3: green) or GFP alone (black).
(TIF)

S1 Movie HMVEC-C cardiac microvascular endothelial cells pattern the Matrigel substrate. Note the co-movement of cells and the gel substrate during the process. Phase contrast time lapse microscopy with 10x objective and 12 h duration. Seeding density was 1600 cells/mm^2^.
(MOV)

S2 Movie 3T3 fibroblast cells pattern the Matrigel substrate. Note the co-movement of cells and the gel substrate during the process. Phase contrast time lapse microscopy with 10x objective and 8 h duration. Seeding density was 1600 cells/mm^2^.
(MOV)

S3 Movie A431 epithelial carcinoma cells pattern the Matrigel substrate. Note the co-movement of cells and the gel substrate during the process. Phase contrast time lapse microscopy with 10x objective and 22 h duration. Seeding density was 1600 cells/mm^2^.
(MOV)

S4 Movie HT29 colon carcinoma cells pattern the Matrigel substrate. Note the co-movement of cells and the gel substrate during the process. Phase contrast time lapse microscopy with 10x objective and 22 h duration. Seeding density was 2400 cells/mm^2^.
(MOV)

S5 Movie HUVEC cells remodel the Matrigel substrate into a network of interconnected vertices and polygonal cell-free areas. Note the co-movement of cells and the gel substrate during the process. Phase contrast time lapse microscopy with 10x objective and 24 h duration. Seeding density was 140 cells/mm^2^.
(MOV)

S6 Movie A431 epithelial carcinoma cells maintain a monolayer when seeded at 100Phase contrast time lapse microscopy with 10x objective and 20 h duration. Seeding density was 2680 cells/mm^2^.
(MOV)

S7 Movie Time development of pattern formation in a simulated system of contractile cells and their ECM microenvironment, represented by spheres, interacting through elastic beams visualized as rods. Note the formation of dense cell nodes and cell-free areas from the initial homogeneous cell layer. Simulation with *N* = 300 particles, *L* = 20*d*_0_ = 500 μm, *k* = 1.5, F*/*F*_0_ = 1.
(MOV)

S8 Movie A431 epithelial carcinoma cell monolayer on Matrigel substrate expands a wound. The wound was punctured, shown by white circle, at 1 h after cell attachment. Note the co-movement of gel substrate with cells. Phase contrast time lapse microscopy with 10x objective and 8 h duration. Seeding density was 1690 cells/mm^2^.
(MOV)

S9 Movie Pattern formation of A431 epithelial carcinoma cell cultures on Matrigel substrate in the presence of 10 μM blebbistatin (top right panel), 30 μM Y27632 (bottom right panel), or in the absence of inhibitors (left panel). Note the smaller pattern size in the right panels. Phase contrast time lapse microscopy with 10x objective and 21 h duration. Seeding density was 1600 cells/mm^2^.
(MOV)

S10 Movie Pattern formation of s100A4 overexpressing A431 epithelial carcinoma cell lines on Matrigel substrate. A431 clones overexpress either the non-functional mutant S100A4 form (A431-mutS100A4, left panel) or the wild type S100A4 (A431-S100A4, right panel). Note the formation of smaller structures in the right panel. Phase contrast time lapse microscopy with 10x objective and 24 h duration. Seeding density was 1600 cells/mm^2^.
(MOV)
