# Supplementary figures and images for: Matrigel patterning reflects multicellular contractility
Source: PLoS Comput Biol. 2019 Oct 25;15(10):e1007431. doi: 10.1371/journal.pcbi.1007431 (PMC6834294; doi:10.1371/journal.pcbi.1007431)

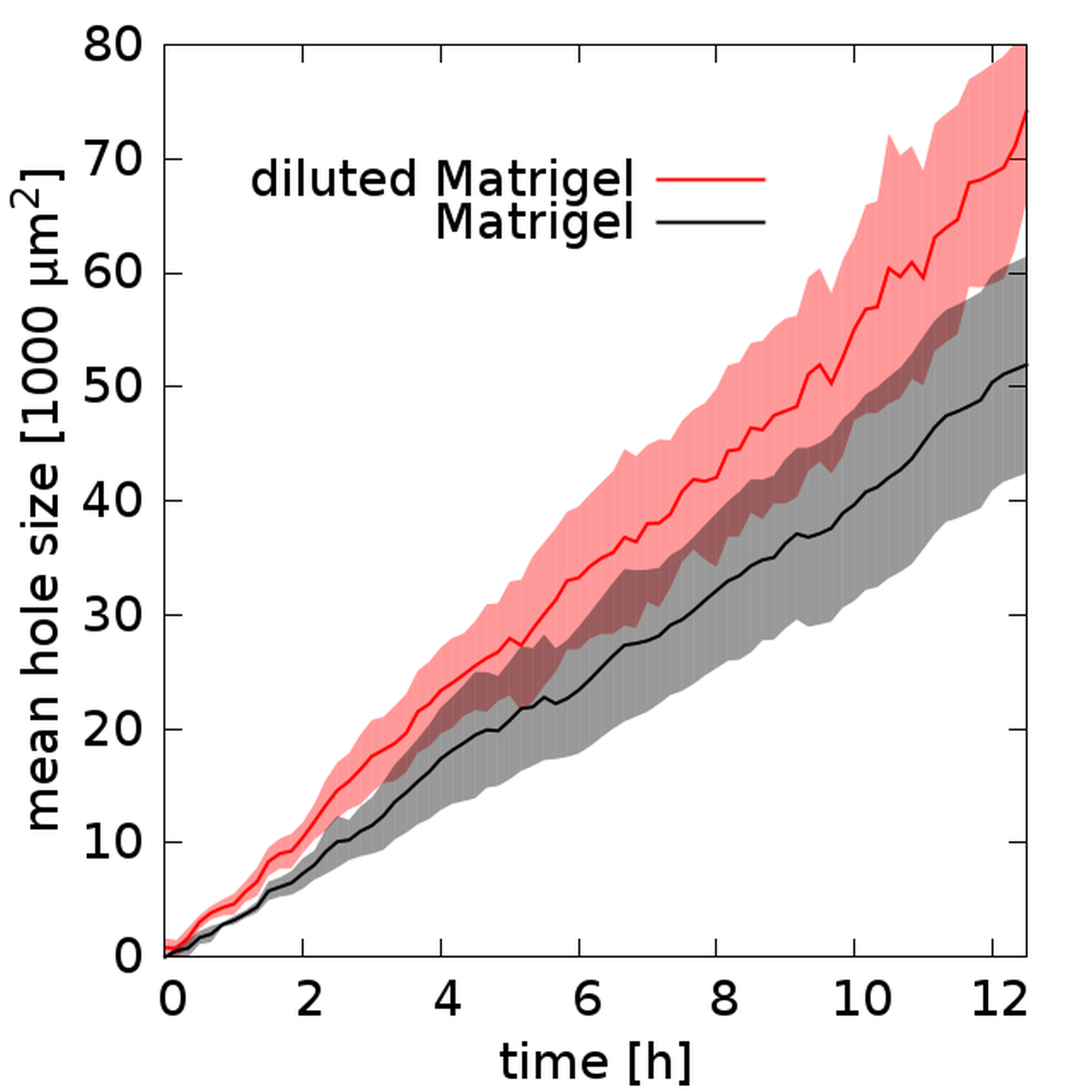

Supplement: S1 Fig — Time dependence of the mean hole size (cell-free area), A¯) of the developing pattern. A431-GFP cells were seeded at 1600 cells/mm2 initial density on Matrigel or Matrigel diluted to 90% v/v with PBS. Error stripes represent SEM, calculated from n = 3 independent sets of experiments. (TIF) [file pcbi.1007431.s001.tif]

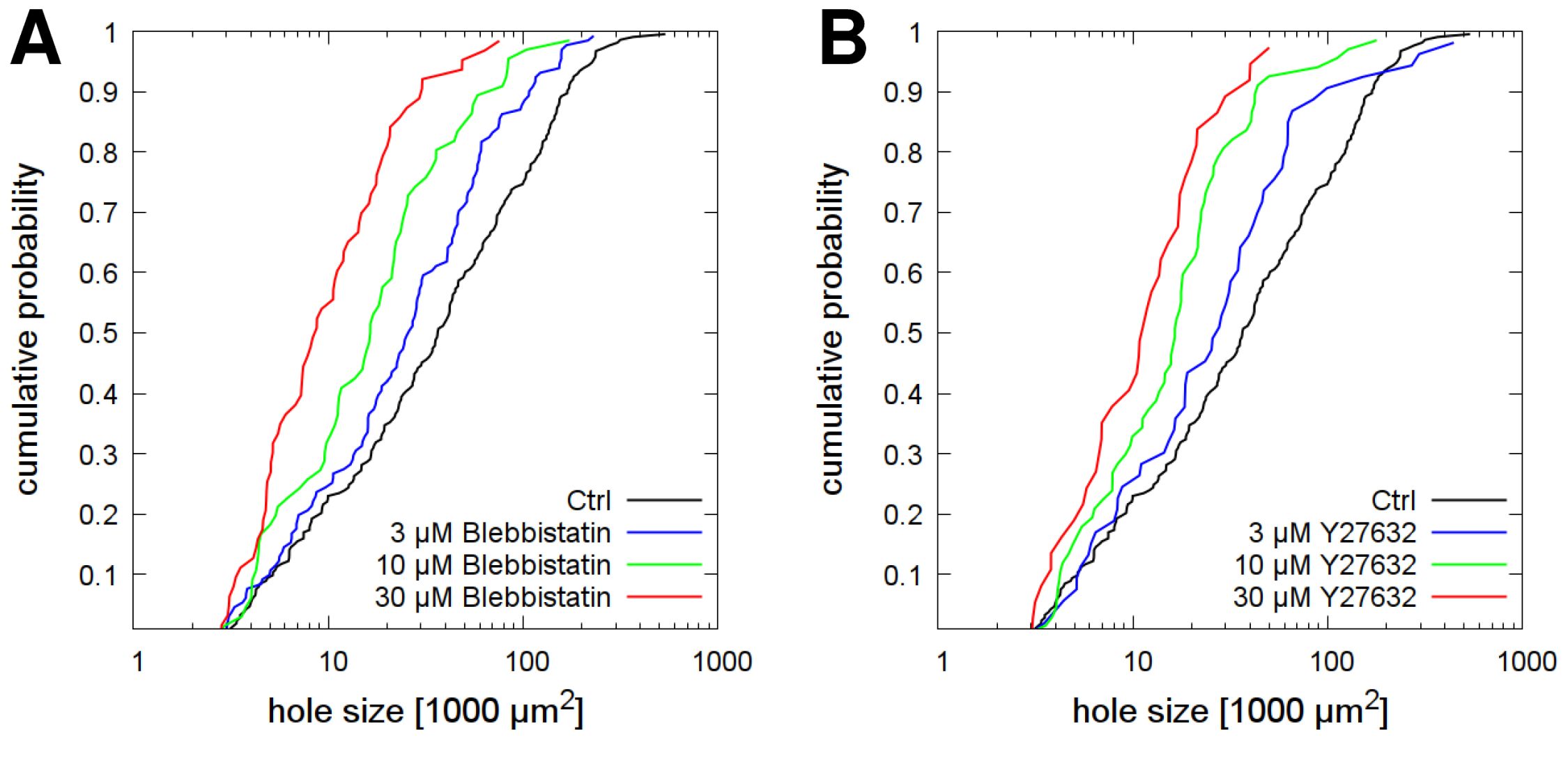

Supplement: S2 Fig — Either blebbistatin (A) or Y27632 Rho kinase inhibitor (B) effectively blocks the formation of large holes in a concentration dependent manner within the Matrigel patterning assay. Cumulative distribution functions of particle free area (hole) sizes indicate the fraction of holes that are larger than the value at the abscissa. Each distribution was pooled from n = 3 sets of microscopic fields, imaged 15 h after seeding A431 epithelial carcinoma cells on Matrigel substrate. (TIF) [file pcbi.1007431.s002.tif]

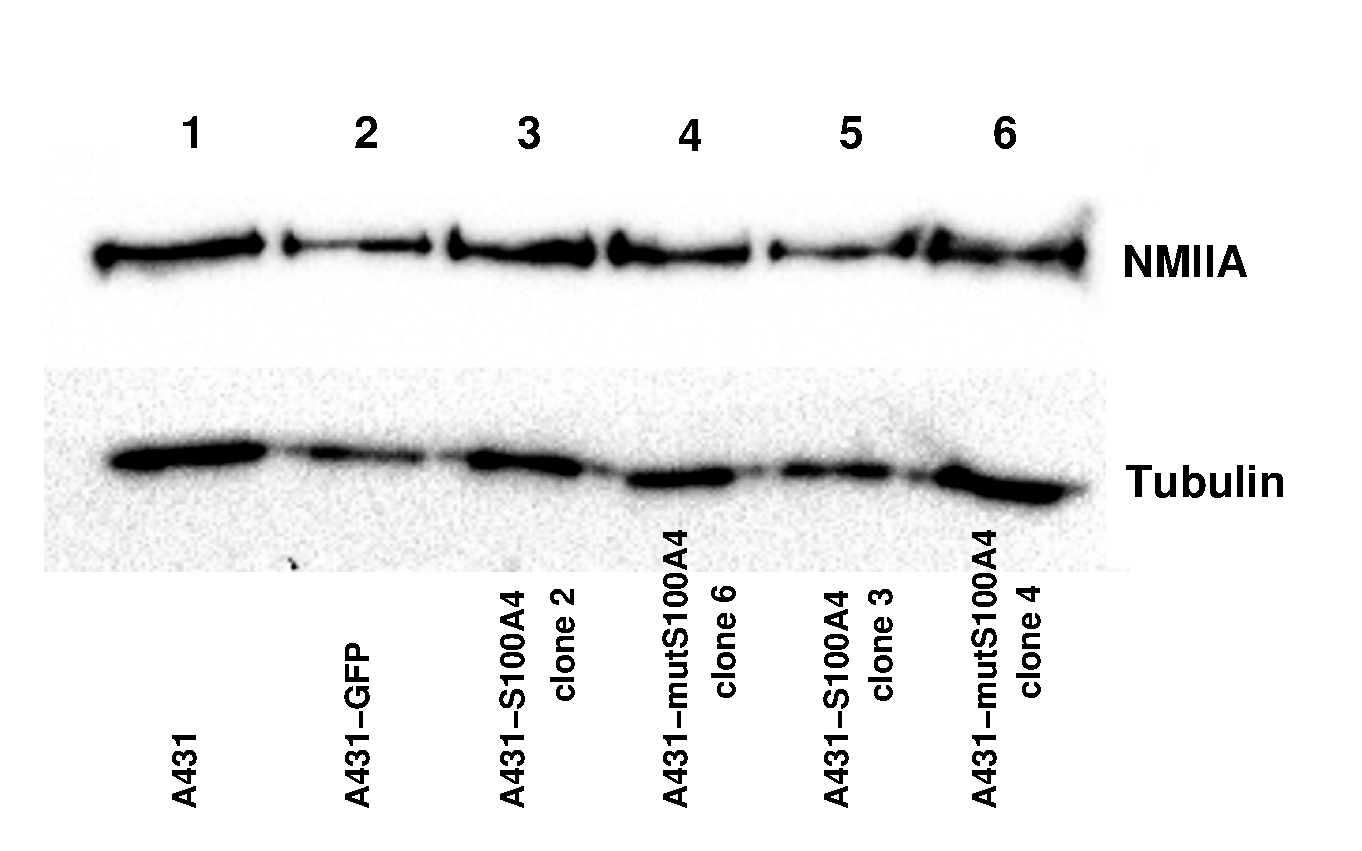

Supplement: S3 Fig — Upper panel: Lysates of A431 cells (lane 1), GFP-expressing A431 cells (lane 2) and clones overexpressing either wild type S100A4 (lanes 3 and 5), or truncated non-functional mutS100A4 (lanes 4 and 6) were immunoblotted for non-muscle myosin II A isoform. NMIIA is present in all clones with a relative molecular mass of around 200 kDa. Lower panel: For loading control, the lower part of the blot was immunolabeled for β-tubulin (50 kDa). (TIF) [file pcbi.1007431.s003.tif]

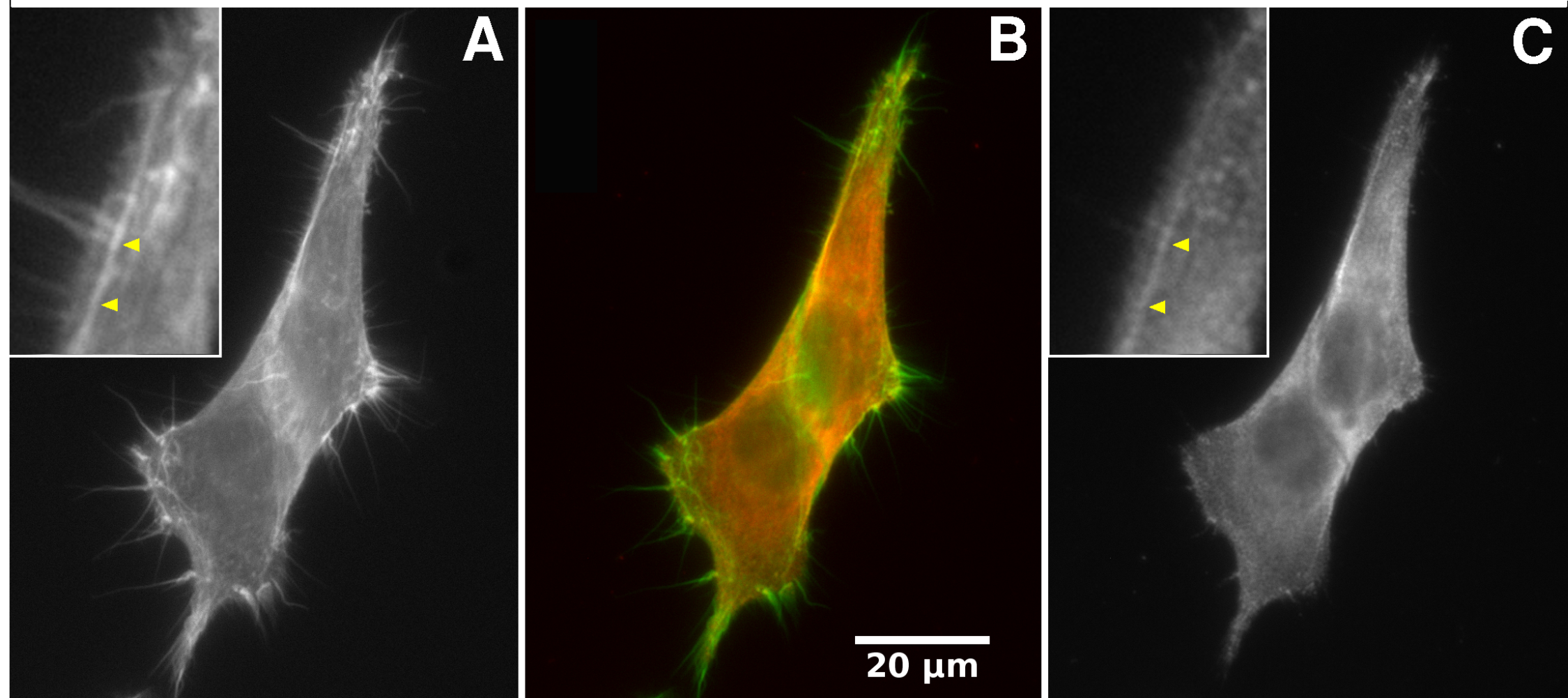

Supplement: S4 Fig — F-actin was visualized by fluorescein-labeled phalloidin (panel A, green in panel B), while NMIIA was immunolabeled (panel C, red in panel B). Colocalization is seen as structures of yellow color (panel B) and pointed to by yellow arrowheads in the insets of panels A and C. Scale bar: 20 μm, 40X objective. (TIF) [file pcbi.1007431.s004.tif]

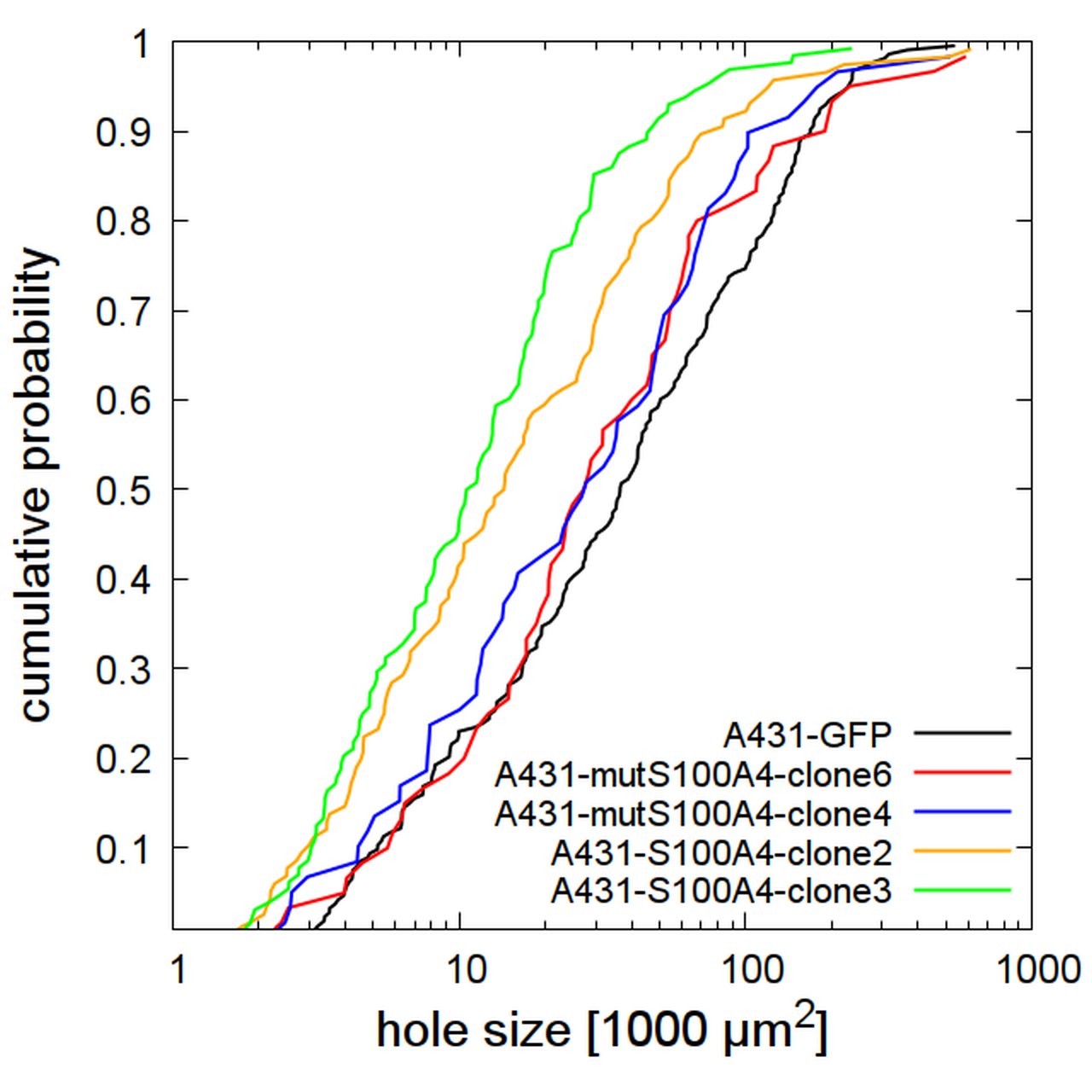

Supplement: S5 Fig — Cumulative distribution functions of particle free area (hole) sizes indicate the fraction of holes that are larger than the value at the abscissa. Each data set was pooled from n = 4 independent sets of microscopic fields, imaged 15 h after seeding A431 epithelial carcinoma cells on Matrigel substrate. A431 clones were either overexpressing a non-functional mutant S100A4 (clone6: red and clone4: blue), the wild type S100A4 (clone2: orange and clone3: green) or GFP alone (black). (TIF) [file pcbi.1007431.s005.tif]
